# Expression and function of the luteinizing hormone choriogonadotropin receptor in human endometrial stromal cells

**DOI:** 10.1038/s41598-022-12495-9

**Published:** 2022-05-21

**Authors:** O. N. Mann, C.-S. Kong, E. S. Lucas, J. J. Brosens, A. C. Hanyaloglu, P. J. Brighton

**Affiliations:** 1 Division of Biomedical Sciences, Clinical Sciences Research Laboratories, Warwick Medical School, University of Warwick, Coventry, CV2 2DX UK; 2 Centre for Early Life, University of Warwick, Coventry, CV4 7AL UK; 3 Tommy’s National Centre for Miscarriage Research, University Hospitals Coventry & Warwickshire NHS Trust, Coventry, CV2 2DX UK; 4 Institute of Reproductive and Developmental Biology, Department of Metabolism, Digestion and Reproduction, Faculty of Medicine, Imperial College London, Hammersmith Campus, Du Cane Road, London, W12 0NN UK

**Keywords:** Reproductive biology, Cell signalling, Pharmacology

## Abstract

The human luteinising hormone choriogonadotropin receptor (LHCGR) is a G-protein coupled receptor activated by both human chorionic gonadotropin (hCG) and luteinizing hormone (LH), two structurally related gonadotropins with essential roles in ovulation and maintenance of the corpus luteum. LHCGR expression predominates in ovarian tissues where it elicits functional responses through cyclic adenosine mononucleotide (cAMP), Ca^2+^ and extracellular signal-regulated kinase (ERK) signalling. LHCGR expression has also been localized to the human endometrium, with purported roles in decidualization and implantation. However, these observations are contentious. In this investigation, transcripts encoding *LHCGR* were undetectable in bulk RNA sequencing datasets from whole cycling endometrial tissue and cultured human endometrial stromal cells (EnSC). However, analysis of single-cell RNA sequencing data revealed cell-to-cell transcriptional heterogeneity, and we identified a small subpopulation of stromal cells with detectable *LHCGR* transcripts. In HEK-293 cells expressing recombinant LHCGR, both hCG and LH elicited robust cAMP, Ca^2+^ and ERK signals that were absent in wild-type HEK-293 cells. However, none of these responses were recapitulated in primary EnSC cultures. In addition, proliferation, viability and decidual transformation of EnSC were refractory to both hCG and LH, irrespective of treatment to induce differentiation. Although we challenge the assertion that LHCGR is expressed at a functionally active level in the human endometrium, the discovery of a discrete subpopulation of EnSC that express *LHCGR* transcripts may plausibly account for the conflicting evidence in the literature.

## Introduction

Human chorionic gonadotropin (hCG) and luteinising hormone (LH), like all other gonadotropin hormones, are heterodimers consisting of two monomeric glycoproteins that combine to make a functional protein. They share a common α-subunit, with structural and functional specificity conferred through differing β-subunits. Despite structural similarities, hCG and LH exhibit different temporal profiles and distinct physiological roles^1^. In women, LH is produced and released by gonadotropic cells of the anterior pituitary gland, and surges during the mid-luteal phase of the menstrual cycle to drive ovulation and initiate the development of the corpus luteum, a temporary ovarian endocrine structure that produces progesterone. hCG, however, is secreted by trophoblast cells of early embryos and the placenta and plays a vital role in sustaining circulating progesterone during early pregnancy^1,2^.

Within the cycling endometrium, signals from the post-ovulatory rise in progesterone converge with local cyclic adenosine monophosphate (cAMP) production to drive a process of differentiation termed decidualization. This transforms endometrial stromal cells (EnSC) into specialized secretory decidual cells and marks the remodelling of endometrial tissue into the decidua of pregnancy, an immune privileged and nutritive matrix that supports embryo implantation and development^3^. However, reprogrammed EnSC can also emerge as acute progesterone-independent senescent decidual cells, which propagate within the tissue via inflammatory paracrine secretions and secondary senescence^4,5^. Thus, as progesterone levels fall in the late luteal phase of a non-conception cycle, the decidual tissue is consumed by sterile inflammation and cellular senescence before eventual breakdown and shedding through menstruation^4–6^. However, progesterone-dependent decidual cells secrete chemo-attractants to recruit and activate uterine natural killer (uNK) cells, the dominant leukocyte population in the endometrium, which then target and eliminate senescent cells through exocytosis and limit the propagation of senescence in a conception cycle^4,5^. Sustained progesterone signalling is thus essential in maintaining the decidua of pregnancy, and it is embryo-derived hCG that prevents programmed atrophy of the corpus luteum to maintain ovarian progesterone production in a conception cycle. This represents an inflection point in the menstrual cycle whereby the fate of the endometrium is principally dependent upon the selection and implantation of embryos that are capable of secreting sufficient hCG and other fitness hormones^7–10^.

Both hCG and LH act through the luteinising hormone choriogonadotrophin receptor (LHCGR), a 7-transmembrane G-protein coupled receptor (GPCR) comprising a large extracellular domain that binds hCG and LH^1,11,12^. Activated LHCGR couples to Gα_s_ G-proteins to initiate the adenylyl cyclase, cAMP and protein kinase A (PKA) signalling pathway^1,11^. It also activates extracellular signal-regulated kinase (ERK) and protein kinase B (PKB/AKT) to drive cell proliferation, differentiation and survival in a variety of cell types^1,13^. In addition, at high hormone and receptor levels, LHCGR couples to Gα_q_ G-proteins to activate phospholipase C and inositol phosphate signalling to increase intracellular Ca^2+^^14,15^.

Congruent with its role in ovulation and corpus luteum formation, LHCGR is prominently expressed in the ovaries^11^, but several studies reported extra-gonadal expression in tissues of the lower reproductive tract, including a variety endometrial cells^16–24^. However, these observations have been challenged^25^. Full-length *LHCGR* transcripts contain 11 exons and 10 introns, but several splice and truncated variants have been identified^26,27^. In one study, full-length *LHCGR* transcripts were absent in 3 out of 8 secretory phase endometrial biopsies and in 10 out of 12 decidua from early pregnancy^17^. They were also non-detectable in a further study except for a variant that lacks an extracellular domain^28^. In addition, many conclusions are derived from immunogenic assays, which are limited by the sensitivity and specificity of antibodies that target gonadotropin hormone receptors^29^.

The functional role of LHCGR in the endometrium is also controversial. For example, there is opposing evidence for hCG-mediated generation of intracellular cAMP^18,22,23,30–32^, for the role of LHCGR in decidual prolactin production^16,30,32,33^, and whether expression and function are cycle-dependent^17,19,21,22,32^.

In the present study, we mined publicly available RNA sequencing data. Transcript for *LHCGR* were either low or undetectable in bulk sequencing datasets from whole endometrial biopsies and cultured primary endometrial stromal cells (EnSC) but were identified in a discrete sub-set of EnSC from single-cell RNA-sequencing datasets. However, primary EnSC cultures were non-responsive to hCG and LH.

## Results

### Lack of *LHCGR* transcripts in the endometrium

**Figure 1:**
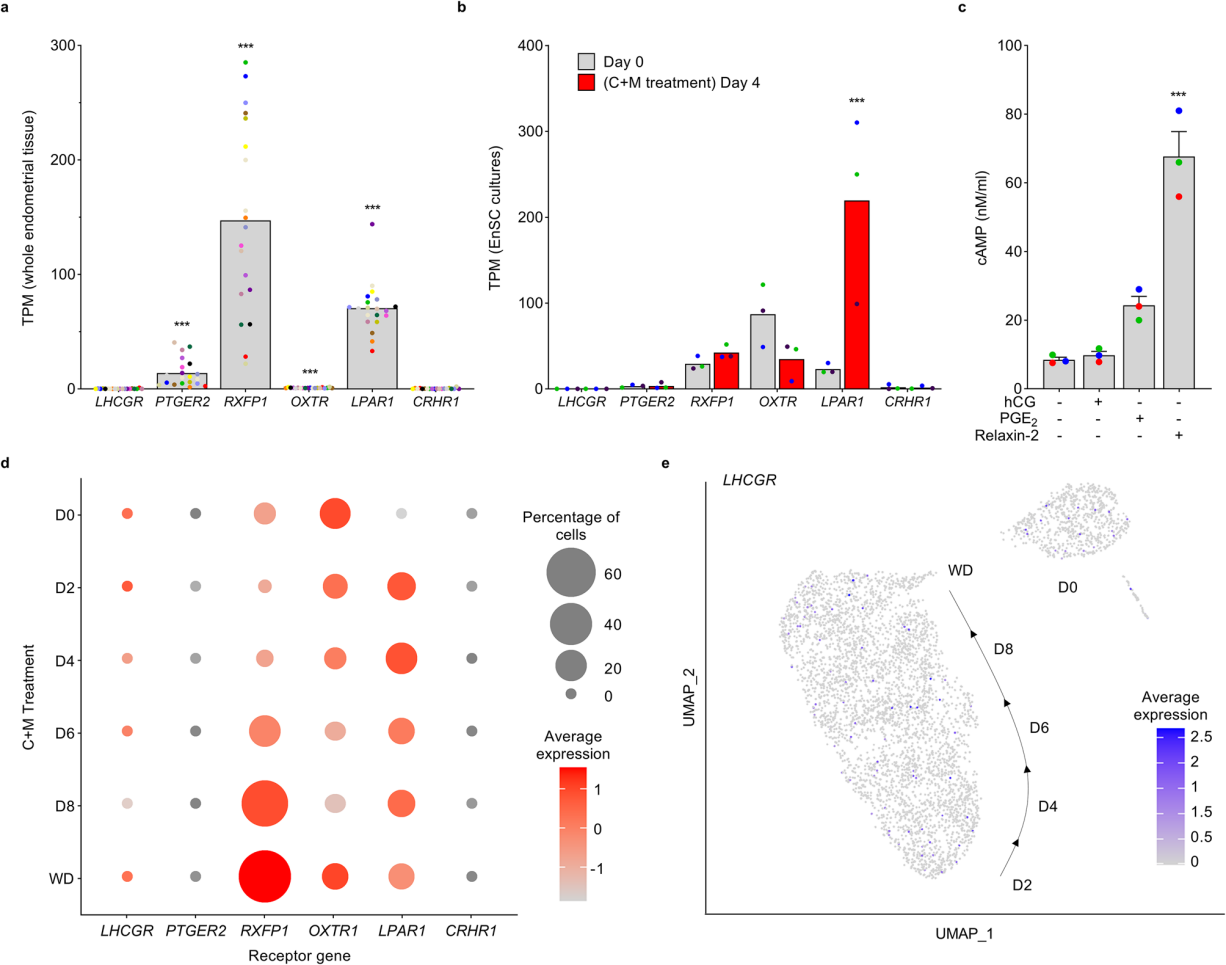
LHCGR expression in whole endometrial biopsies and EnSC. (**a**) Expression of genes coding GPCRs within whole endometrial biopsies (GEO GSE65102). Data points from individual patients are colour matched and shown together with bar graphs denoting mean (n = 20). Significance was determined by ANOVA and Dunnett’s multiple comparison test using *LHCGR* as the comparator. ***P < 0.001. (**b**) Expression of GPCRs in undifferentiated EnSC (day 0) and cells decidualized with 8-br-cAMP and MPA (C + M) for 4 days (GEO GSE104721). Individual data points from 3 biological replicates are shown with bar graphs denoting mean. Significance was determined by two-way ANOVA and Sidak’s multiple comparison test, ***P < 0.001. (**c**) cAMP responses in EnSC following stimulation with hCG, PGE_2_ and Relaxin-2. Significance was determined by ANOVA and Dunnett’s multiple comparison test using *LHCGR* as the comparator, ***P < 0.001. (**d**) Dot-plot to visualize scRNA-seq data (GEO GSE127918) showing the percentage of EnSC expressing GPCRs, and the Log_2_-transformed average expression within these cells. (**e**) UMAP (uniform manifold approximation and projection) plot of *LHCGR* expression within scRNA-seq data from undifferentiated (D0) EnSC and those treated with C + M for time points indicated (D = days) before withdrawal (WD). Transcriptomic profiling to identify cell states is shown in Supplementary Fig. S1.

A summary of the literature pertaining LHCGR expression in endometrial cells is shown in Table 1. To explore LHCGR expression in endometrium, we mined publicly available RNA-sequencing (RNA-seq) data from whole endometrial biopsies and primary EnSC cultures (GEO accession numbers: GSE65102 and GSE104721). In whole tissues, transcripts encoding *LHCGR* were rare or undetectable. Average levels were 0.15 ± 0.26 (mean ± SD) transcripts per million (TPM) and significantly lower (*P* < 0.05) than those encoding other GPCRs implicated in endometrial regulation, including the prostaglandin E_2_ (PGE_2_) receptor (*PTGER2*), relaxin family peptide receptor-1 (*RXFP1*) and the lysophosphatidic acid receptor (*LPAR1*) (Fig. 1a). Equally, in EnSC, *LHCGR* transcripts were rare or absent, whereas those encoding other GPCRs including *PTGER2*, *RXFP1*, *LPAR1* and the oxytocin receptor (*OXTR*), were expressed prominently (Fig. 1b). For functional comparison, EnSC were challenge with 10 nM hCG, 1 µM PGE_2_ or 1 µM Relaxin-2 for 5 min to assess activation of Gα_s_-coupled LHCGR, prostanoid EP_2_ (*PTGER2*) and relaxin family peptide receptor 1 (*RFXP1*) receptors, respectively. The relative levels of cAMP mirrored those of receptor transcripts, with LHCGR unable to raise intracellular levels above basal (Fig. 1c). The use of comparator GPCRs provides perspective on the low level of *LHCGR* transcripts, and although each receptor will have a unique activation and regulation signature, there was a strong correlation between transcript expression and functional responses.

**Table 1:** Expression of LHCGR in endometrial cells and tissue.

Refs.	Cell source	Cell types	*n*	Phase of cycle	Detection method	Antibody source	LHCGR localisation	LHCGR MW
^16^	Primary cells	Stromal	21	Proliferative, decidualisation induced	Western Blot Northern Blot [^125^I]-hCG-binding	Raised against amino acids 15–38	–	80 kDa
^28^	Primary tissue from premenopausal hysterectomy samples	All	25	Proliferative, luteal, other	RT-PCR	–	Extracellular sequence not detected Fragments found in 19/25 samples	-
^17^	Primary tissue from premenopausal hysterectomy samples	All	30	Proliferative, early, mid and late luteal	Nested RT-PCR	–	Full length receptor in proliferative, early and mid luteal biopsies Detected in 5/8 late luteal biopsies and 2/12 early decidua	-
^18^	HES cell line	Epithelial	–	–	[^125^I]-CG crosslinking RT-PCR Western Blot	Unspecified	–	80 kDa
^19^	Primary cells	Epithelial	6	Proliferative, luteal	RT-PCR	–	Detected in epithelial cells from luteal phase Lower levels in proliferative phase	–
^20^	HES cell line	Epithelial	–	–	Western blot	Raised against amino acids 257–271	–	80 kDa
^21^	In vivo	All	67	Proliferative, early, mid and late luteal, pregnant	IHC	Raised against exon 9	Glandular epithelium, spiral arteries, stroma	–
	Primary cells, HES cell line	Epithelial	2	–	ICC	#sc-26341	Plasma membrane, occasionally nuclear after 5 IU/ml hCG treatment	–
^22^	Primary endometrial tissue	All	22		Western blot	unspecified	–	68 kDa
^23^	Primary cells	Stromal	46	Mid-luteal	RT-qPCR ICC	#sc-25828	Transcript variants *a* and *b* detected Plasma membrane and perinuclear	–
^24^	Primary cells	All	12	Proliferative	ICC RT-PCR	#sc-25828	Transcripts present in all endometria tested Plasma membrane, glandular epithelium	–

One disadvantage of bulk RNA-sequencing is that it masks subpopulation variability and heterogeneity between cells. Single-cell RNA sequencing (scRNA-seq) enables molecular characterisation of individual cells, and is a powerful tool to overcome these limitations by revealing heterogeneity within cell populations^34^. We therefore mined scRNA-seq data (GEO accession number: GSE127918) that reconstructed the decidual pathway by treating EnSC with 8-bromo-cAMP (cAMP) and the progestin medroxyprogesterone (MPA) over 8 days. Cells clustered into distinct cell states based on their transcriptional profiles in a time-dependent manner (Supplementary Fig. S1). Irrespective of cell state, the average expression of *LHCGR* transcripts was low when compared to *RXFP1*, *LPAR1* or *OXTR*. One notable observation was the considerable transcriptional heterogeneity within EnSC for all receptors, including *LHCGR* (Fig. 1d). Out of the 4574 cells in the dataset we identified a discrete population of 97 (2.1%) cells where transcripts encoding *LHCGR* were detected (Fig. 1e). In addition, the major cell types within whole endometrial biopsies (GEO accession number: GSE127918) were deconvolved and identified based on their transcriptomic profile (Supplementary Fig. S1), and consistent with EnSC, *LHCGR* transcripts were detected in a subpopulation of stromal and epithelial cells (Supplementary Fig. S1). To further substantiate the heterogeneity in whole tissue we mined independent single cell transcriptomic data^35^, and again observed *LHCGR* transcripts in a small population of stromal cells (Supplementary Fig. S2).

### hCG and LH preparations are bioactive

**Figure 2:**
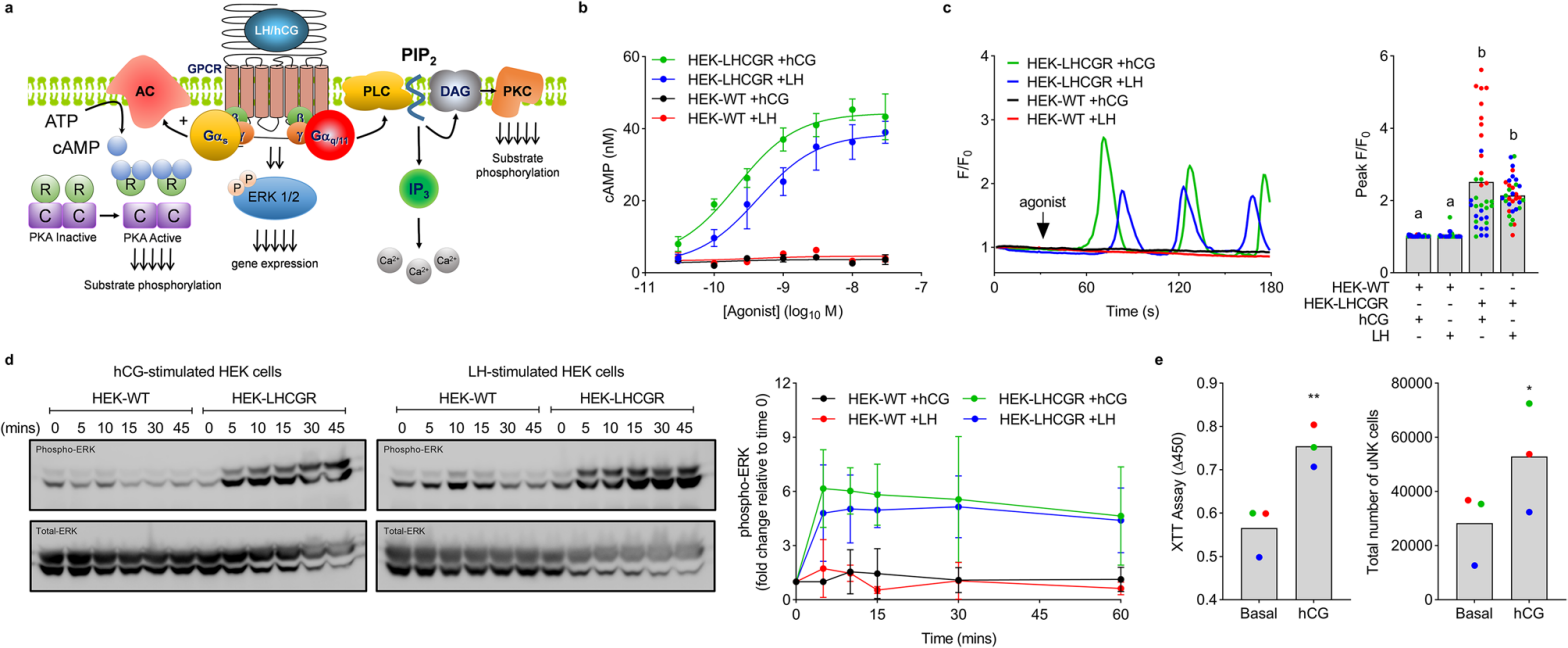
hCG and LH are bioactive. (**a**) Schematic representation of signalling pathways downstream of LHCGR. (**b**) Concentration-dependent cAMP responses to hCG and LH in HEK-WT and HEK-LHCGR cells. (**c**) Representative Ca^2+^ traces in HEK-WT and -LHCGR cells after treatment with 1 µM hCG or LH. Data are shown as fold change in fluorescence relative to time point 0 (F/F_0_) and represent one individual cell. Mean peak responses from 36 cells analysed from 3 separate cultures (denoted as separate colours) are shown in the right panel with differing letters indicating significance between groups (*P* < 0.05), (ANOVA and Tukey’s multiple comparison test). (**d**) Representative western blots showing levels of phospho-ERK in HEK-WT and -LHCGR cells challenged with either 10 nM hCG or LH (left panels). Levels of phospho-ERK were normalized to total-ERK and shown as a fold change relative to unstimulated cells (time point 0) (right panel). Data are mean ± SD, n = 3. (**e**) Impact of 10 nM hCG on uNK cell proliferation as measured by XTT assay (left panel) and cell counting (right panel). Individual data points from 3 biological replicates are shown with bars denoting mean values. Significance was determined by paired t-test with *P < 0.05 and **P < 0.01.

Previous studies on the responsiveness of endometrial cells to LH and hCG are contentious, as summarized in Table 2. Figure 2a depicts how active LHCGR couples to Gα_s_ G-proteins and activates adenylate cyclase to increase intracellular cAMP, but the receptor can also trigger Ca^2+^ release and activate kinase pathways including ERK. To validate gonadotropin bioactivity, these key signalling pathways were investigated in HEK-293 cells recombinantly expressing LHCGR (HEK-LHCGR). Cells were challenged with either hCG or LH and compared to parallel experiments in wild-type HEK-293 cells (HEK-WT). Robust increases in intracellular cAMP were detected in HEK-LHCGR cells challenged for 5 min with either hCG or LH. Inductions were concentration-dependent and generated pEC_50_ values of − 9.671 (213 pM) for hCG and − 9.370 (426 pM) for LH, with maximal responses achieved at ~ 10 nM (Fig. 2b). By contrast, HEK-WT cells did not respond. HEK cells were also loaded with the Ca^2+^ sensitive dye Calbryte-520 and challenged with high concentrations of hCG or LH (1 µM). Both agonists elicited robust Ca^2+^ responses in HEK-LHCGR cells, but not HEK-WT cells (Fig. 2c). Further, a robust and sustained induction of phosphorylated (active)-ERK was detected with both ligands in HEK-LHCGR, whereas HEK-WT cells did not respond (Fig. 2d).

**Table 2:** Functional activation of LHCGR in primary endometrial cells.

Ref	Cell source	Cell types	*n*	Phase of cycle	hCG source	Concentration hCG type	LHCGR response	% change in response (relative to control)
^85^	Primary cells	Stromal	27	Proliferative	National Hormone and Pituitary Program	1.49–14,900 IU/ml hCG type unknown	Cox-2 protein levels by Western Blot	100% increase
							Cox-2 transcript levels by Northern blot	Information not given, only statistically significant above 149 IU/ml
^31^	Primary cells	Stromal	17	Luteal	Sigma, #994F-0137	50, 250, or 500 ng hCG type unknown	cAMP concentration by radioimmunoassay	500% increase
^86^	Primary cells	Stromal	21	Proliferative	National Hormone and Pituitary Program	149 IU/ml hCG type unknown	LHCGR transcript levels by Northern blot	60% decrease
							LHCGR expression by Western blot	40% decrease
^87^	Primary cells	Stromal	20	Secretory	Sigma	10 nM hCG type unknown	PRL expression via RT-qPCR	60% decrease in control patients 20% increase in RPL patients
							PROK1 expression via RT-qPCR	50% decrease in control patients 350% increase in RPL patients
^88^	Primary cells	Stromal	5	–	Source not specified	0.1–100 IU/ml hCG type unknown	PRL via RT-qPCR and ELISA	58% decrease with in mRNA and protein
							IGFBP1 via RT-qPCR and ELISA	50% decrease in mRNA and protein
^16^	Primary cells	Stromal	24	Proliferative	National Hormone and Pituitary Program	1.49–149 IU/ml hCG type unknown	Morphology by phase contrast microscopy	Morphological changes consistent with decidualisation
							PRL protein levels by radioimmunoassay	No change, but 200% increase in combination with E_2_ + P_4_
^32^	Primary cells	Stromal	68	Follicular, periovulatory, early-late luteal	Teikokuzouki Pharmaceuticals	0.01–100 IU/ml hCG type unknown	PRL protein levels by radioimmunoassay	100% decrease at 100 IU/ml. No change at 1, 10 IU/ml
							cAMP concentration by radioimmunoassay	66% decrease at 100 IU/ml. No change at 1, 10 IU/ml
^18^	HES cell line	Epithelial	–	–	National Hormone and Pituitary Program	1.8 IU/ml Recombinant hCG	cAMP concentration by ELISA	Statistically insignificant
							phospho-ERK by Western blot	Increased band intensity (not quantified)
							PGE_2_ production by ELISA	100% increase
^19^	Primary cells	Epithelial	28	Proliferative, luteal	Pregnyl, Organon	1–50 IU/ml Urinary hCG	LIF production by ELISA	100–125% increase
							IL-6 production by ELISA	20% decrease
							Cell proliferation by BrdU ELISA	Statistically insignificant
^89^	Primary cells	Epithelial	18	Proliferative, luteal	Pregnyl, Organon, Ovitrelle, Serono	5–50 IU/ml Recombinant hCG	VEGF protein levels by ELISA	60% increase
^20^	HES cell line	Epithelial	–	–	EMD Serono	10 IU/ml Recombinant hCG	phospho-ERK by Western blot	400% increase
^90^	Primary cells	Epithelial	15	Proliferative, luteal	National Hormone and Peptide Program	0.2–20 IU/ml Recombinant hCG	FGF2 protein levels, among others, by multiplex immunoassay	50% increase
^21^	Primary cells HES cell line	Epithelial	2	– –	National Hormone and Pituitary Program	20 IU/ml (acute) or 0.5–5 + 20 IU/ml (chronic) Recombinant hCG	phospho-ERK by Western Blot	40–50% increase
							Transepithelial resistance	40% decrease (acute dose)
							Cell adhesion	20–50% increase
^22^	Primary cells	All	22	Various	Source not specified	10 μg/ml hCG type unknown	Adenylyl cyclase activity by radioactive cAMP production	Statistically insignificant
^23^	Primary cells	Stromal	46	Mid-luteal	Sigma (#CG10)	10 IU/ml Urinary hCG	cAMP concentration by ELISA	100% increase
							phospho-ERK by Western blot	100% increase
^40^	Primary cells	Stromal	5	Proliferative	Profasi, Serono	1–10 IU/ml Urinary hCG	Proliferation by [^3^H]-Thymidine incorporation	65% reduction
^24^	Primary cells	All	12	Proliferative	Luveris, Merck	0–100 ng/ml Recombinant LH	cAMP concentration by ELISA	200% increase
							CYP191A and P450ssc expression by RT-qPCR	300% and 200% increase, respectively

In addition to LHCGR, LH and hCG also bind with mannose and other C-type lectin receptors^36–39^. Significantly, hCG-mediated mannose receptor activation has been shown to induce uNK cell proliferation^38^. To demonstrate alternative hCG-receptor interactions, and to provide further evidence of bioactivity, we investigated the proliferative effects of hCG on uNK cells isolated from non-pregnant endometrial biopsies. We demonstrated previously that these uNK cells are viable, and stain positively for both the pan-leukocyte marker CD45 and the uNK specific marker CD56^9^. In this study, uNK cell proliferation, as quantified by XTT assay, was augmented by 133 ± 35% (*P* = 0.0093) (mean ± SD) after 2 days in culture with 10 nM hCG. The overall number of uNK cells, as determined via haemocytometer, also increased by 202 ± 54% (*P* = 0.030) when cultured with hCG (Fig. 2e). These observations prompted us to characterize the expression profiles of mannose receptors within the endometrium. First, we mined publicly available RNA-seq data from endometrial biopsies (GEO accession number: GSE65102) and found that transcripts for both the mannose receptor C-type 1 (*MRC1*) and type 2 (*MRC2*) were abundant, and ~ 48 (*P* < 0.0001) and ~ 443 (*P* < 0.0001) fold higher than those for *LHCGR*, respectively (Supplementary Fig. S2). Next, we examined expression in EnSC (GEO accession number: GSE104721) and in isolated uNK cells (GEO accession number: GSE159288) and observed high levels of transcripts for *MRC2*, low levels for *LHCGR* and none for *MRC1* (Supplementary Fig. S2). Likewise, *MRC1* transcripts were absent within our whole tissue scRNA-seq data (GEO accession number: GSE127918), whereas *MRC2* transcripts were abundantly expressed in the stromal compartment and immune cells, including lymphocytes (Supplementary Fig. S2). Similar observations from independent scRNA-seq data also indicated an absence of *MRC1* transcripts in most cell types except a small population of macrophages, but widespread *MRC2* expression in stromal and immune cells (Supplementary Fig. S2). Interestingly, for all receptors there was conspicuous heterogeneity between individual cells.

### EnSC do not respond to hCG or LH

**Figure 3:**
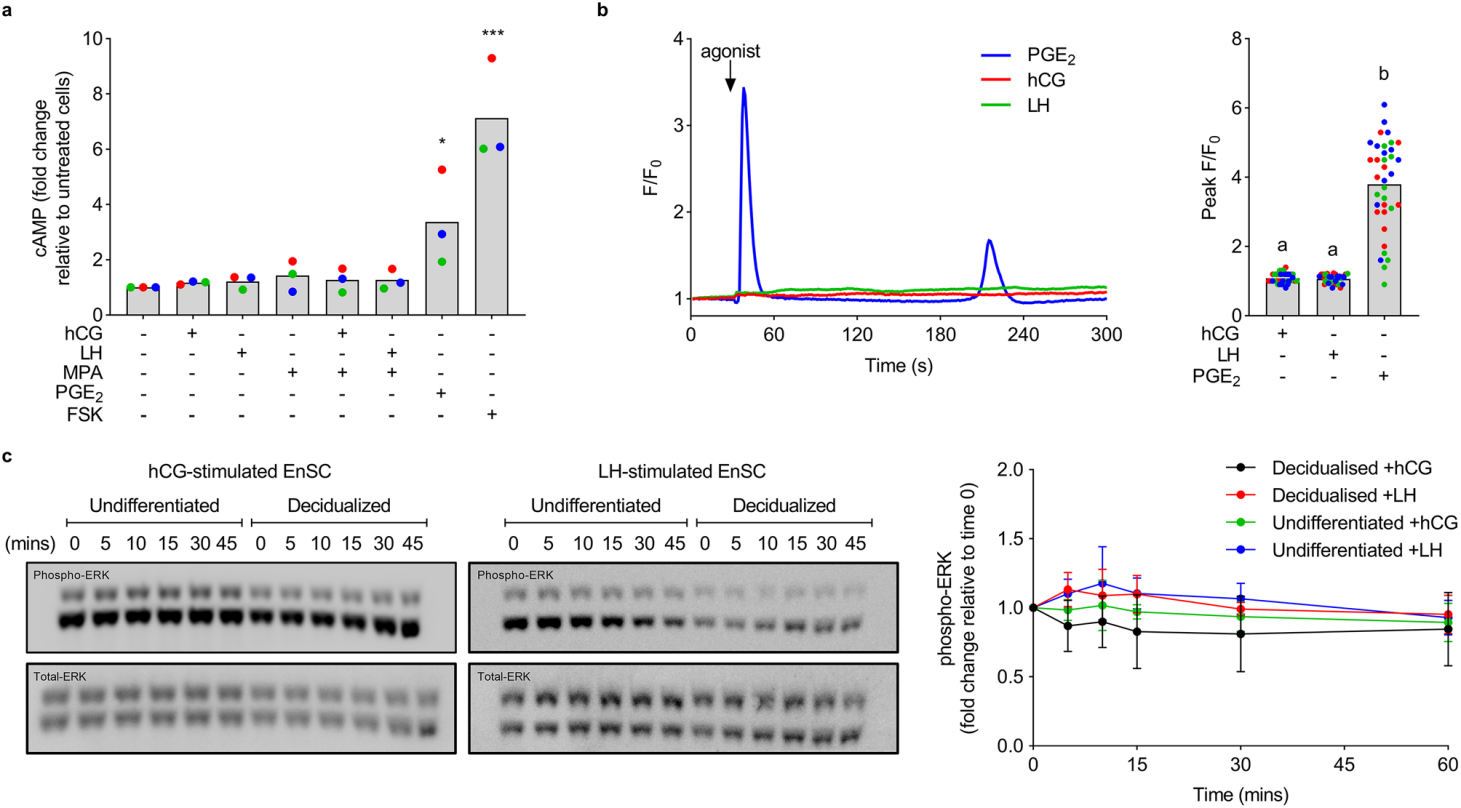
Lack of LHCGR signalling in EnSC. (**a**) The relative change in cAMP following 5-min stimulation with 10 nM hCG or LH (with or without MPA), 2 µM PGE_2_ or 5 µM forskolin (FSK). Data are shown from 3 independent primary cultures, with bars denoting mean values. Significance was determined by ANOVA and Dunnett’s multiple comparison test using unstimulated cells as the comparator. *P < 0.05 and ***P < 0.001. (**b**) Representative Ca^2+^ traces from EnSC challenged with 1 µM hCG or LH, or 2 µM PGE_2_ (left panel). Data are shown as fold change in fluorescence relative to time 0 (F/F_0_) and represent one individual cell. Mean peak responses of 36 cells from 3 independent cultures (denoted as separate colours) are shown in the right panel. Different letters indicate statistical differences (*P* < 0.05) between groups (ANOVA and Tukey’s multiple comparison test). (**c**) Representative western blots showing levels of phospho-ERK in undifferentiated and decidualized EnSC following stimulation with either 10 nM hCG or LH (left panels). Levels of phospho-ERK were normalized to total-ERK and shown as fold change relative to unstimulated cells (time point 0) (right panel). Data are mean ± SD from 3 individual primary cultures. All changes from time 0 are non-significant (ANOVA and Dunnett’s multiple comparison test).

We next sought to investigate hCG and LH-mediated signalling in EnSC. In contrast to responses in HEK-LHCGR, neither hCG nor LH increased intracellular cAMP in primary EnSC cultures. Cells were however responsive to PGE_2_ (*P* = 0.037), known to activate Gα_s_-coupled EP_2_/EP_4_ prostanoid receptors, and forskolin (*P* < 0.0001), the receptor-independent adenylate cyclase activator (Fig. 3a). Further, the same concentration of hCG and LH (1 µM) that elicited robust Ca^2+^ responses in HEK-LHCGR cells failed to increase intracellular Ca^2+^ in EnSC, despite strong responses to PGE_2_, likely activating the Gα_q/11_-coupled EP_1_/EP_3_ prostanoid receptors (Fig. 3b). Likewise, basal levels of phosphorylated-ERK in both undifferentiated EnSC and those decidualized for 8 days were unchanged following stimulation with either hCG or LH (Fig. 3c).

### Lack of functional LHCGR effects in EnSC

**Figure 4:**
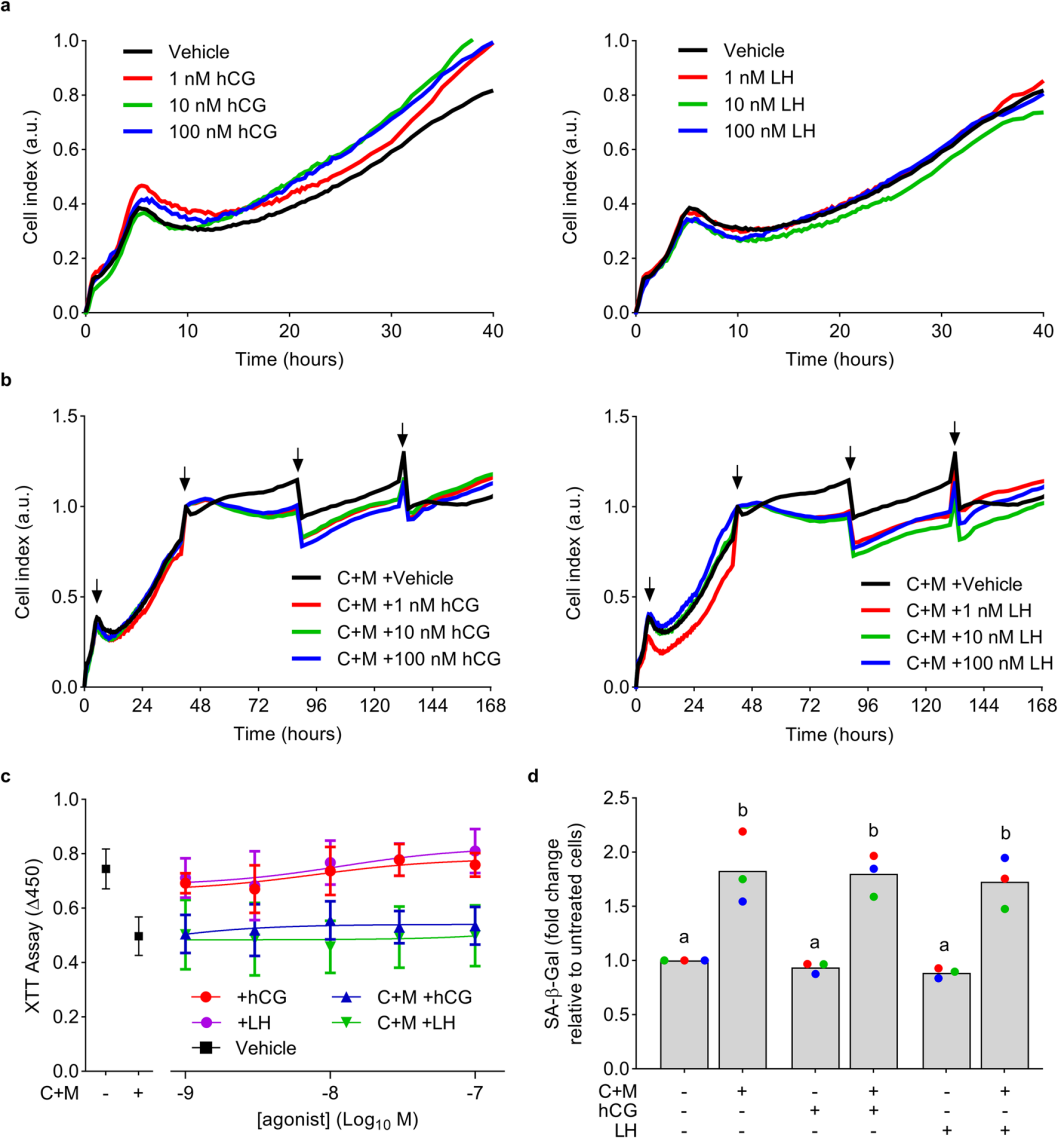
EnSC proliferation and viability are refractory to LHCGR ligands. (**a**,**b**) Impact of varying concentrations of hCG (left panels) and LH (right panels) on the proliferation of undifferentiated (**a**) or decidualizing (**b**) EnSC as quantified by xCELLigence. Traces indicate changes in impedance (cell index) from time 0. Arrows indicate media changes. Data are representative of 3 independent primary cultures, with average data non-significant by ANOVA and Dunnett’s multiple comparison test using vehicle as the comparator. (**c**) Impact of a range of concentrations of hCG and LH on cell proliferation in undifferentiated and decidualized (C + M) EnSC, as measured by XTT assay. Data are mean ± SD from 3 independent primary cultures. All concentrations of hCG and LH are non-significant via ANOVA and Dunnett’s multiple comparison test. (**d**) Effects of 10 nM hCG and LH on the induction of SA-β-Gal, a marker of cellular senescence. Data from 3 individual primary cultures are shown with different letters above the bars (mean values) indicating statistical differences (*P* < 0.05) between groups (ANOVA and Dunnett’s multiple comparison test).

hCG inhibits proliferation in EnSC but also protects against stress-induced apoptosis^33,40,41^. Anti-apoptotic effects are also observed in ovarian cells, but conversely here LHCGR activation is pro-proliferative^1^. To screen for these key physiological responses, EnSC were grown on microelectronic sensors (xCELLigence) to permit real-time monitoring of cultures using electrical impedance to represent changes in cell shape and confluency. EnSC cultures exhibited a bi-phasic pattern of growth that stabilized into linear and sustained proliferation after 2 days. Neither the pattern, magnitude, nor rate of proliferation were affected by hCG or LH, even at high concentrations (1 µM) (Fig. 4a). Similarly, no differences in impedance were observed with hCG or LH during continuous monitoring of EnSC as they decidualized (C + M treatment) for 8 days (Fig. 4b). Furthermore, there were no differences in cell viability and proliferation, as measured by XTT assay, after 2-day culture with either ligand, even up to concentrations of 10 µM (Fig. 4c). The emergence of cellular senescence in a subpopulation of stromal cells is a key characteristic of decidualization^4,5^. We therefore investigated whether LH or hCG effected the induction of senescence-associated β-Galactosidase (SA-β-Gal), a key biomarker of cellular senescence. As expected, levels of SA-β-Gal in 8 day-decidualized EnSC cultures increased 1.83 ± 0.33-fold (mean ± SD) compared to untreated cultures. However, neither hCG (1.80 ± 0.19) nor LH (1.73 ± 0.23) impacted on SA-β-Gal activity (Fig. 4d).

**Figure 5:**
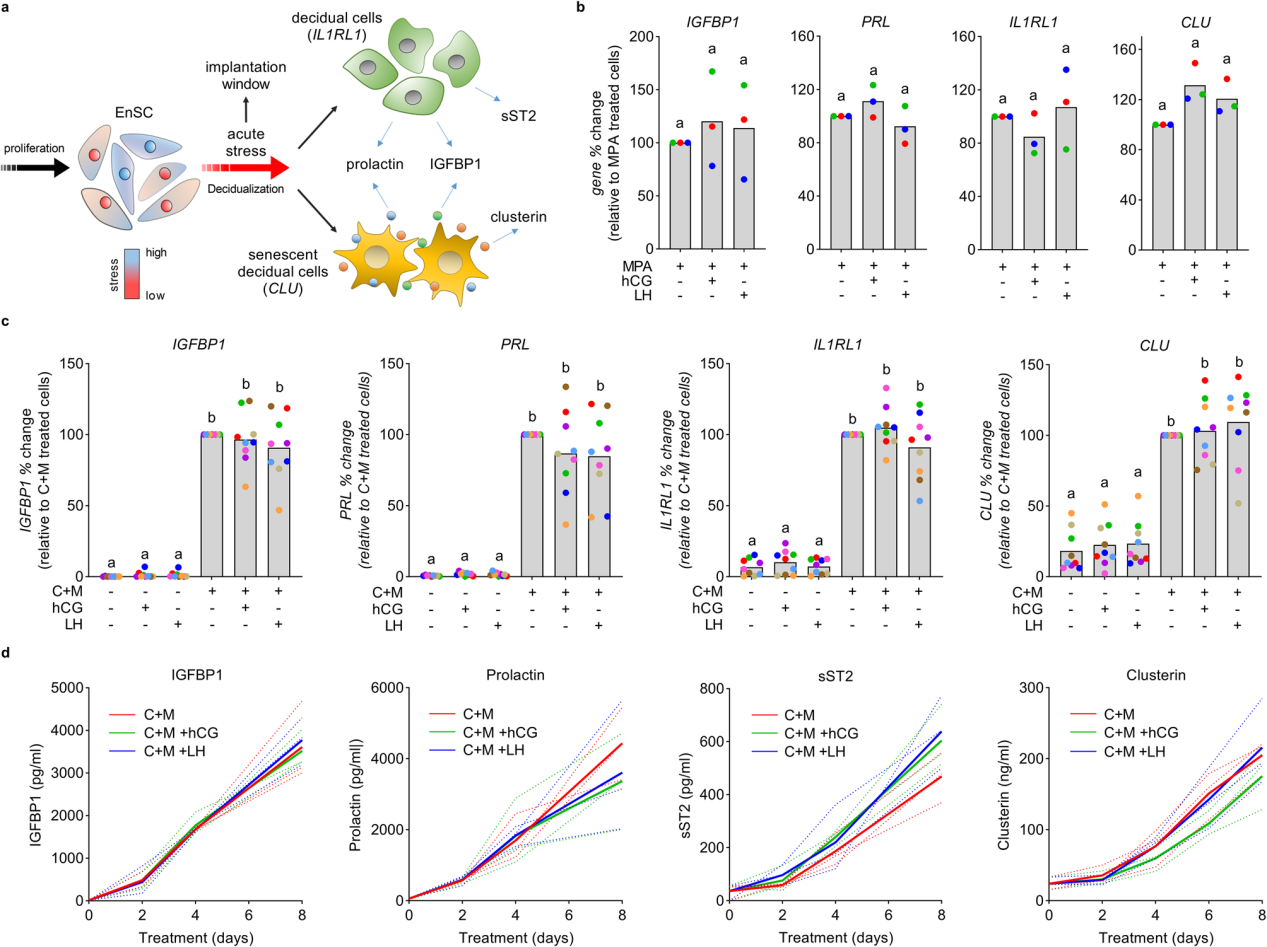
hCG and LH have no effect on EnSC decidualization. (**a**) Schematic representation of the emergence of decidual and senescent decidual cells and the genes and secreted factors associated with each population. Modified with permission from Kong et al.^9^. (**b**) RT-qPCR analysis of markers for decidualization (*IGFBP1* and *PRL*) and decidual (*IL1RL1*) and senescent (*CLU*) EnSC after 8-day treatment with 10 nM hCG or LH in combination with MPA. (**c**) Impact of 10 nM hCG and LH on the induction (C + M treatment) of decidual (*IGFBP1* and *PRL*) and population specific (*IL1RL1* and *CLU*) marker genes (n = 9). Data are shown as individual biological replicates from independent cultures with bars denoting mean values. Different letters indicate statistical differences (*P* < 0.05) between groups (ANOVA and Dunnett’s multiple comparison test). (**d**) Impact of 10 nM hCG and LH on the decidual induction (C + M) of secreted factors in EnSC. Plots from individual patients are shown with bold lines indicating mean. Data are non-significant via two-way ANOVA and Dunnett’s multiple comparison test using C + M as the comparator.

Several studies propose a role for LHCGR in decidualization (see Table 2). Thus, we investigated whether hCG and LH regulate the induction and secretion of key decidual markers. As depicted in Fig. 5a, the expression of canonical decidual markers *PRL* and *IGFBP1* co-insides with the emergence of markers that differentiate senescent and decidual cells. *IL1RL1* is a specific biomarker of decidual cells and encodes both the transmembrane (ST2L) and soluble (sST2) interleukin 1 receptor like-1, an anti-inflammatory decoy receptor that sequesters IL-33. *CLU* on the other hand codes the molecular chaperone protein clusterin (apolipoprotein J) and is enriched within the senescent subpopulation^4^. First, since convergence of sustained cAMP and progesterone signalling is essential for decidualization^3^, we treated EnSC continuously for 8 days with MPA in combination with hCG or LH. In keeping with an absence of an acute rise in cAMP levels, sustained activation of LHCGR had no impact on either the induction of decidual markers (*PRL* and *IGFBP1*) or the emergence of decidual subpopulations (*IL1RL1* and *CLU*) (Fig. 5b). To further investigate the role of LHCGR in EnSC differentiation, cultures from 9 individual patients were decidualized with for 8 days in combination with hCG or LH. Both the average basal and induced levels of decidual genes were refractory to hCG and LH (Fig. 5c), however considerable variability between cultures was observed for all genes. In addition, we found no significant changes in secreted levels of prolactin, IGFBP1, clusterin or sST2 when cells were decidualized for 8 days in the continued presence of LHCGR ligands (Fig. 5d).

## Discussion

hCG secretions from early embryos indirectly influence the endometrium by preventing atrophy of the corpus luteum, thereby sustaining circulating progesterone and maintaining the decidua of pregnancy^3^. The direct effects of hCG however, are poorly understood. When hCG is flushed through the human or baboon uterus, changes in endometrial gene expression associated with implantation and decidualisation are observed, including upregulation of PRL, IGFBP1, leukaemia inhibitory factor (LIF), MMPs and IL-6^42,43^. Although this suggests hCG-dependent activation of the LHCGR is important at the embryo-endometrial interface, attempts at recapitulating these observations in EnSC are contentious. Here, we sought to elucidate the responsiveness of EnSC to LHCGR ligands but failed to detect activation of key signal pathways or functional cellular responses. However, we identified for the first time, a subpopulation of stromal cells that do express *LHCGR* transcripts, and which may plausibly contribute to the discord between studies.

Although defining a threshold where genes can be considered functionally active poses a challenge for the analysis of transcriptomic sequencing data, there is a consensus that genes with TPM < 2 are unlikely to be actively transcribed^44–46^. Mean values for *LHCGR* transcripts in whole endometrial tissues were 0.15 TPM and should therefore be considered non-expressed. However, insight from scRNA-seq data highlights heterogeneity in *LHCGR* expression, and the identification of a subpopulation of EnSC with detectable *LHCGR* transcripts. Unfortunately, *LHCGR* expressing cells did not cluster as a distinct population, and thus comparative profiling with non-expressing cells was not performed. Similar cellular heterogeneity for other GPCRs does trigger cell-specific responses to inflammatory cues in vascular and inflammatory cells^47,48^, but our current understanding of GPCR heterogeneity in the endometrium is rudimentary, and whether LHCGR-positive cells are relevant in pathological conditions such as infertility, miscarriage and endometriosis awaits elucidation. The responsiveness of our primary EnSC to deciduogenic stimulus in the presence of hCG or LH varied markedly between cultures, and differential expression profiles may be a key factor in the diversity of responses that we, and others, observe.

The functional analysis of LHCGR is further complicated by the complex levels of regulation that govern its expression and function^49^. For example, several splice variants including non-functional receptors have been identified^26–28^, as have immature and constitutively active receptors^11,50,51^, plasma membrane clusters^50,52–54^, follicular stimulating hormone receptor (FSHR) heterodimers^55,56^, and receptor density-dependent signalling^57–59^. Fluctuations in expression and G-protein coupling have also been linked to circadian rhythms^60^. In addition, LHCGR is known to undergo rapid but transient agonist-mediated down-regulation via a LHCGR binding protein (LRBP) that accelerates the degradation of receptor mRNA^61–63^. Further, like many other GPCRs, the LHCGR undergoes desensitisation and internalisation upon activation that not only regulates G protein signalling, but also activates additional signalling pathways^64,65^. The consequences of these splice variants and regulatory mechanisms is poorly understood, and may impact profoundly on the expression and function of the receptor.

Unlike LH, CG is an evolutionarily newer protein arising from a gene duplication event of the LHβ gene and is unique to primates^66,67^. Both covalently bind carbohydrates, but hCG has more moieties on the β subunit creating a wider variety of potential glycoforms. Indeed, LH and hCG differentially activate the LHCGR to drive distinct biological activities^1,68^. The glycosylation status of hCG is likely to be heterologous and will change depending on the source of hCG and stage of pregnancy, such as the hyperglycosylated variant that predominates in early pregnancy^2,69,70^. Commercially available formulations are purified by a variety of methods and differ in their source and biological composition. Consequently, variations in glycosylation patterns between preparations are observed that differentially activate LHCGR and display unique biological activity^69,71–74^. While not addressed directly in this study, as neither LH nor hCG forms employed induced functional responses, the source and glycosylation status of gonadotropins should be considered when interpreting and comparing experimental observations.

The role of glycosylation is complicated further by non-LHCGR mediated effects through mannose and other lectin-like receptors. These receptors recognize terminal mannose, N-GlcNAc and fructose residues on glycans attached to proteins and are known to interact with pituitary hormones include TSH and LH^36–38^. They are abundant in a range of immune cells, many of which, including uNK cells, macrophages, dendritic cells, and regulatory T cells, infiltrate the maternal decidua. In this study, hCG augmented uNK cell proliferation, a response linked previously to activation of mannose receptors^38^. There is also further evidence that hCG stimulates cytokine release from peripheral blood mononuclear cells (PBMC), including IL-18, IL-1β, IL-6, and TNF-α, interferon γ and soluble IL-2 receptor^36,75–77^. In many cases, responses were sensitive to excess D-mannose and refractory to deglycosylated hCG, emphasising the importance of glyco-conjugates. Given the critical function of uNK cells and cytokine release during decidualisation and implantation^4,5^, immune cell/lectin receptor activation cannot be excluded from the endometrial responses to uterine hCG flushing. Here, we showed that stromal and immune cells predominantly express the type 2 mannose receptor (*MRC2*), whereas the *LHCGR* was mostly absent. It is therefore necessary for researchers to design future experiments that deplete cells of LHCGR and mannose receptors to identify receptor activation.

In an evolutionary context, hCG is no longer considered a useful signal of embryo fitness^78^. Considering the excessive production in early pregnancy, its extended half-life and multiple glycoforms, the role of hCG-mediated activation of the LHCGR in the endometrium, at least from the evolutionary perspective of embryo selection, may have become redundant. However, the endometrium undergoes profound adaptations during pregnancy in response to embryonic and environmental cues^3^. How the activation and regulation of endometrial LHCGR is modified during pregnancy, especially in response to embryonic hCG, is poorly understood, and highlights the need for models that recapitulate the multicellular endometrial environment during early pregnancy.

In summary, we report that transcripts for LHCGR were largely absent in non-pregnant endometrial tissues and EnSC cultures but were identified in a small subpopulation of stromal cells. Both hCG and LH failed to activate key signalling cascades and functional responses in primary EnSC. Our findings, highlights the need for caution when interpreting data that ignores both heterogeneity within endometrial cell populations and non-canonical hCG-LHCGR signalling through mannose and C-type lectin receptors.

## Methods

### In silico analysis

Receptor expression data were derived from in silico analysis of RNA sequencing data within the Gene Expression Omnibus (GEO) repository. Expression from bulk RNA-sequencing within secretory phase whole endometrial biopsies was derived from GEO accession number GSE65102^79^, in undifferentiated EnSC and cells decidualized for 4 days from GSE104721^80^, and in isolated uNK cells from GSE159266^81^. To determine receptor expression within individual cells we analysed single-cell RNA-sequencing (scRNA-seq) data from whole endometrial biopsies obtained 8 and 10 days following the pre-ovulation LH surge, and from EnSC cultures decidualized for 8 days (GSE127918)^4^. Single cell expression within endometrial tissues was also derived from an independent data set^35^, which was visualized using Cellxgene, a web-based interface for viewing single-cell transcriptomic data^82^.

### Endometrial biopsy collection

*Ethical approval and consent: *Endometrial biopsies were obtained from women attending the Implantation Research Clinic at the University Hospitals Coventry and Warwickshire National Health Service Trust. The collection of endometrial biopsies for research was approved by the NHS National Research Ethics—Hammersmith and Queen Charlotte's & Chelsea Research Ethics Committee (REC reference: 1997/5065) and Tommy’s National Reproductive Health Biobank (REC reference: 18/WA/0356). Written informed consent was obtained prior to tissue collection in accordance with the Declaration of Helsinki, 2000. *Endometrial sampling:* Endometrial biopsies were collected 6–10 days after the pre-ovulatory LH surge using a Wallach Endocell™ Endometrial Cell Sampler and were processed immediately. A total of 40 biopsies were used for this investigation and patient demographics are shown in Supplementary Table S1. *EnSC dissociation and culture:* EnSC were isolated from fresh biopsies using methods described previously^83^. In summary, cells were dissociated by mincing and enzymatic digestion of the extracellular matrix using 500 µg/ml collagenase type 1a (Merck Life Sciences UK Ltd) and 100 µg/ml DNAse I (Lorne Laboratories Ltd, Reading, U.K) for 1 h at 37 °C. Glandular clumps and debris were removed by passing digested tissue through a 40 µM cell strainer before centrifugation (400×*g*, 5 min). Pellets were re-suspended and cultured in DMEM/F12 media supplemented with 10% dextran-coated charcoal stripped Foetal Bovine Serum (DCC-FBS), antibiotic–antimycotic solution (10 U/ml penicillin, 10 µg/ml streptomycin and 0.25 µg/ml Amphotericin B (Thermo Fisher Scientific)), 2 mM l-glutamine, 1 nM E_2_ and 2 mg/ml insulin at 37 °C in a 5% CO_2_, humidified environment. Growth media was refreshed within 18 h to remove non-adherent cells, including immune cells, red blood cells and cellular debris. uNK cells were isolated from this supernatant as required (see below). For passage and seeding, EnSC were lifted with 0.25% trypsin, and all treatment/stimulation protocols were performed in phenol-free DMEM/F‐12 media culture containing 2% DCC‐FBS and antibiotics/antimycotics. *Cell treatment*: To induce differentiation, EnSC were decidualized with 0.5 mM 8‐bromo‐cAMP (cAMP) and 10 µM medroxyprogesterone acetate (MPA) (C + M treatment) as required for individual experiments. Cell were treated with hCG and LH using concentrations and protocols required for individual experiments.

### hCG and LH preparations

Urinary hCG was obtained from Merck Life Sciences UK Ltd (#CG10) (Gillingham, U.K), with an activity of 16 IU/mg. At a concentration of 10 nM, this activity equates to 5.82 IU/ml. Human LH was supplied by A.F. Parlow, from the National Hormone and Peptide Program, Harbor-UCLA Medical Center. LH activity was 6100 IU/mg, and 10 nM equates to 1.59 IU/ml.

### HEK-293 cell culture

FLAG-human LHCGR plasmids were constructed and stably transfected into Human Embryonic Kidney (HEK)-293 cells as described previously^84^. HEK-LHCGR cells were maintained in DMEM supplemented with 10% DCC-FBS, 1% penicillin/streptomycin and 400 µg/ml Geneticin at 37 °C in a 5% CO_2_, humidified environment. Geneticin was excluded from wild-type HEK (HEK-WT) cells. HEK cells were passaged using 0.05% trypsin (< 1 min) and seeded into wells pre-coated with 50 µg/ml poly-d-lysine to aid adherence. Prior to assay, cells were downregulated in serum-free media for 18–24 h.

### cAMP assay

Changes in cellular cAMP levels were determined using a modified 2-step High-Throughput Time Resolved Fluorescence (HTRF) cAMP kit (Cisbio Bioassay, Codolet, France). Confluent HEK or EnSC cultures in 6-well plates were down-regulated prior to stimulation and washed and assayed in Krebs–Henseleit Buffer (KHB) (composition: NaCl: 118 mM, d-glucose: 11.7 mM, MgSO_4_·7H_2_O: 1.2 mM; KH_2_PO_4_ 1.2 mM, KCl 4.7 mM, HEPES 10 mM, CaCl_2_·2H_2_O: 1.3 mM, pH 7.4, 37 °C), containing 300 µM IBMX. Cells were challenged with hCG or LH (or appropriate controls) for 5 min at 37 °C and lysed by rapid aspiration of buffer and immediate addition of 50 µl ice-cold lysis buffer. Cell lysates were collected from wells and cAMP determined on a PHERAstar FS plate reader (BMG Labtech Ltd, Ortenberg, Germany) as per manufacturer’s instructions.

### Ca^2+^ assay

HEK or EnSC cultures were seeded onto 35 mm glass-bottomed culture dishes (MatTek Corporation, MA, USA) and grown until ~ 80% confluent. Cells were down-regulated overnight, washed in KHB and loaded with 10 µM Calbryte™ 520 AM (Cal-520) (Stratech Scientific Ltd, Ely, U.K.), a Ca^2+^ sensitive fluorescent dye, for 1 h at 37 °C in a 5% CO_2_ humidified environment. Cal-520 was removed prior to assay for 15 min to allow de-esterification of AM esters. Dishes were mounted onto the stage of a µManager-controlled Olympus IX-85 microscope and equilibrated at 37 °C in KHB before imaging. Cells were challenged with 1 µM hCG or LH by direct bath addition and Cal-520 fluorescence captured by a DC4100 LED stack and GFP filter set (ThorLabs, Newton, NJ, U.S.A.). Images were obtained using a Zyla sCMOS camera (Andor Technology Ltd, Belfast, U.K.) at a frequency of 1 Hz for up to 5 min. Videos were analysed in ImageJ where changes in cytosolic fluorescence were used as an index of intracellular Ca^2+^. Changes in fluorescence were related to fluorescence at time 0 s to give a fold increase of fluorescence/fluorescence at time 0 s (F/F_0_). Mean peak response data was obtained from 12 cells chosen at random from each independent primary culture.

### Western blot

EnSC and HEK cultures were grown to ~ 80% confluency in 6-well plates and down-regulated for 24 h prior to assay. Where required, EnSC were decidualized for 8 days prior to assay. Cells were assayed in KHB at 37 °C and challenged with 10 nM hCG or LH for up to 45 min. Experiments were terminated by aspiration of buffer and rapid lysis in ice-cold RIPA buffer containing cOmplete mini protease inhibitors (Roche, Basel, Switzerland) and phosphatase inhibitor cocktail 2 (1:1000) (Merck Life Sciences U.K. Ltd). Cell debris was removed from lysates via centrifugation (10,000×*g*, 2 min, 4 °C). Samples were prepared in 25% (v:v) NuPage LDS × 4 sample buffer (Fisher Scientific, Loughborough, U.K.) and 100 nM DTT (Merck Life Sciences U.K. Ltd), and heated at 95 °C for 5 min. Proteins were separated using standard SDS-PAGE electrophoresis techniques on 10% acrylamide gels and transferred onto 0.45 μm nitrocellulose membranes (GE Healthcare, Amersham, U.K.) where non-specific binding was blocked by 5% (w:v) powdered milk in TBS-T (50 mM Tris, 150 mM NaCl, pH 7.4, 0.5% (v:v) Tween-20) for 1 h at RT. Membranes were probed with antibodies targeting phosphorylated-ERK (1:2000 in TBS-T, phospho-p44/42 MAPK (ERK1/2) (Thr202/Tyr204) Antibody #9101, Cell Signalling Technologies, MA, U.S.A.) overnight at 4 °C. Blots were washed clear of unbound antibody in TBS-T before addition of anti-rabbit-HRP secondary antibody (1:1000 in 5% milk/TBS-T; Agilent Technologies, CA, U.S.A.) for 1 h at RT. Blots were again washed, before visualization of immune-reactive bands using ECL reagent (GE Healthcare) and auto-radiography. Protein loading was controlled by uniform cell seeding but also verified by detection of total-ERK. Here, antibodies were stripped from blots by submersion in boiling water for 5 min and re-blocked in 5% milk/TBS-T. Blots were probed with antibodies targeting total-ERK (1:2000 in TBS-T, p44/42 MAPK (ERK1/2) Antibody #9102, Cell Signalling Technologies). The density of individual bands for phospho-ERK was determined using GeneTools gel analysis software (Syngene, Cambridge, U.K.), and expressed relative to levels of total-ERK.

### XTT cell viability assay

EnSC were grown in 96-well plates and down-regulated for 24 h prior to a 2-day treatment with hCG or LH, both with and without C + M treatment to induce decidualization. The viability of EnSC after treatment was assessed using the CyQuant™ XTT cell viability assay (Fisher Scientific) as per manufacturer’s instructions.

### uNK cell isolation and counting

uNK cells were isolated and cultured as described elsewhere^5,83^. Briefly, unattached cells from freshly digested endometrial biopsies were collected following overnight culture and red blood cells were depleted via Ficoll-Paque density medium centrifugation (400×*g*, 30 min, RT). Cell suspensions were pooled from 4–5 patients to increase yield and incubated with magnetic microbeads targeting the uNK specific antigen CD56 (#130-050-401, Miltenyi Biotec, Bergisch Gladbach, Germany) diluted 1:20 in wash buffer (1% BSA/PBS) for 15 min at 4 °C. CD56^+^ cells were isolated through magnetic activated cell sorting (MACS). Isolated uNK cells were cultured in DMEM/F12 media supplemented with 10% DCC-FBS, 1% antibiotic/antimycotic and 125 pg/ml recombinant IL-15, and never assayed beyond day 4. Cells were treated with 10 nM hCG for 48 h before cell viability and proliferation was quantified by XTT assay and cell counting using a Neubauer-improved haemocytometer.

### xCELLigence

EnSC viability and proliferation was assessed in real-time using an xCELLigence® Real Time Cell Analysis (RTCA) DP instrument (ACEA Biosciences Inc, San Diego, CA, U.S.A.). EnSC were seeded in E-16 xCELLigence plates at a density of 10,000 cells/well and cultured for 24 h. Both undifferentiated and decidualizing cells (C + M treated) were monitored in the presence of hCG and LH with electrical impedance referenced to 0 shortly before initiation of treatment. Changes in impedance were captured and analysed using the RTCA Software v1.2 to reflect changes in cell adherence and proliferation and were expressed as the arbitrary unit ‘cell index’.

### Senescence associated-β-galactosidase

Levels of the cellular senescence marker Senescence Associated-β-Galactosidase (SA-β-Gal) were determined using a modified version of the 96-well Quantitative Cellular Senescence Assay kit (Cell Biolabs Inc; CA, U.S.A.). Both undifferentiated and decidualizing (C + M treated) EnSC in 96-well plates were treated with 10 nM hCG or LH for 8 days, with media refreshed every 2 days. To determine levels of SA-β-Gal, cells were washed in ice-cold PBS and lysed in 50 μl ice-cold assay lysis buffer containing cOmplete™ mini protease inhibitors. Lysates (25 µl) were transferred to black-walled, black-bottomed 96-well plates where an equal volume of 2 × assay buffer was added as per manufacturer’s guidelines. Plates were sealed to avoid evaporation and the reaction incubated for 1 h at 37 °C in a non-humidified, non-CO_2_ incubator. The reaction was terminated by 200 μL stop solution and fluorescent intensity units (F.I.U.) were determined on a PHERAstar FS plate reader at 360/465 nm. Assays were normalized by identical cell seeding.

### RT-qPCR

Total RNA from EnSC was extracted using STAT-60 (AMSBio, Abingdon, U.K.), according to manufacturer’s instructions, with recovered RNA quantified on a Nanodrop spectrophotometer. Equal amounts of total RNA were transcribed into cDNA using the QuantiTect Reverse Transcription Kit (Qiagen, Manchester, U.K.) as per manufacturer’s instructions. Target gene expression was analysed using Quantifast SYBR Green Master Mix (Fisher Scientific) on a 7500 Fast Real-Time PCR System (Applied Biosystems, CA, U.SA.). The expression level of each gene was calculated using the ΔCt method and normalized against levels of *L19* housekeeping gene. Primer sequences used were: *PRL* sense 5’-AAG CTG TAG AGA TTG AGG AGC AAA C-3’, *PRL* antisense 5’-TCA GGA TGA ACC TGG CTG ACT A-3’, *IGFBP1* sense 5’-CGA AGG CTC TCC ATG TCA CCA-3’, *IGFBP1* antisense 5’-TGT CTC CTG TGC CTT GGC TAA AC-3’, *IL1RL1* sense 5’-TTG TCC TAC CAT TGA CCT CTA CAA-3’, *IL1RL1* antisense 5’-GAT CCT TGA AGA GCC TGA CAA-3’, *CLU* sense 5’-GGG ACC AGA CGG TCT CAG-3’, *CLU* antisense 5’-CGT ACT TAC TTC CCT GAT TGG AC-3’; *L19* sense 5’-GCG GAA GGG TAC AGC CAA-3’, *L19* antisense 5’-GCA GCC GGC GCA AA-3’.

### ELISA

Supernatant from cell cultures was collected every 2 days and cleared of cellular debris by centrifugation (275×*g*, 5 min, RT) before storage at −20 °C. Levels of secreted IGFBP1, prolactin, clusterin and soluble ST2 (sST2) in culture supernatant were quantified using DuoSet ELISA kits (Biotechne, MN, U.S.A.) as per manufacturer’s instructions. Absorbance at 450 nm was determined on a PHERAstar FS plate reader and concentrations were interpolated using a 4-parameter fit in GraphPad Prism (v9.0).

### Statistical analysis

GraphPad Prism v9.0 (GraphPad Software Inc. CA, U.S.A.) was used for statistical analyses with data presented as fold-change relative to the most informative comparator. Paired Student’s *t*-test was performed to determine statistical significance between 2 groups, and one-way ANOVA, followed by Tukey’s or Dunnett’s post-hoc test for multiple comparisons involving more than 2 groups. Data with 2 independent variables was analysed using a two-way ANOVA (mixed methods) and Dunnett’s multiple comparison test using the most appropriate control as the comparator. In all cases, *P* < 0.05 was considered significant.

## Supplementary Information

Supplementary Information.

## Data Availability

The authors declare that all data supporting the findings of this study are available within the article and its supplementary information files. The datasets analysed during the current study are available in the Gene Expression Omnibus (GEO) repository GEO accession numbers GSE65102^79^, GSE104721^80^, GSE127918^4^ and GSE159266^81^. These were derived from the following public domain resource: https://www.ncbi.nlm.nih.gov/geo.

## References

[1] Casarini L, Santi D, Brigante G, Simoni M (2018). Two hormones for one receptor: Evolution, biochemistry, actions, and pathophysiology of LH and hCG. Endocr. Rev..

[2] Fournier T, Guibourdenche J, Evain-Brion D (2015). Review: hCGs: Different sources of production, different glycoforms and functions. Placenta.

[3] Gellersen B, Brosens JJ (2014). Cyclic decidualization of the human endometrium in reproductive health and failure. Endocr. Rev..

[4] Lucas ES (2020). Recurrent pregnancy loss is associated with a pro-senescent decidual response during the peri-implantation window. Commun. Biol..

[5] Brighton PJ (2017). Clearance of senescent decidual cells by uterine natural killer cells in cycling human endometrium. eLife.

[6] Critchley HOD, Maybin JA, Armstrong GM, Williams ARW (2020). Physiology of the endometrium and regulation of menstruation. Physiol. Rev..

[7] Ewington LJ, Tewary S, Brosens JJ (2019). New insights into the mechanisms underlying recurrent pregnancy loss. J. Obstet. Gynaecol. Res..

[8] Haig D (2019). Cooperation and conflict in human pregnancy. Curr. Biol..

[9] Kong CS (2021). Embryo biosensing by uterine natural killer cells determines endometrial fate decisions at implantation. FASEB J..

[10] Brosens JJ (2014). Uterine selection of human embryos at implantation. Sci. Rep..

[11] Ascoli M, Fanelli F, Segaloff DL (2002). The lutropin/choriocrctnadotropin receptor, a 2002 perspective. Endocr. Rev..

[12] Xie YB, Wang HY, Segaloff DL (1990). Extracellular domain of lutropin choriogonadotropin receptor expressed in transfeced cells binds choriogonadotropin with high-affinity. J. Biol. Chem..

[13] Choi J, Smitz J (2014). Luteinizing hormone and human chorionic gonadotropin: Origins of difference. Mol. Cell. Endocrinol..

[14] Breen SM (2013). Ovulation involves the luteinizing hormone-dependent activation of G(q/11) in granulosa cells. Mol. Endocrinol..

[15] Gilchrist RL, Ryu KS, Ji IH, Ji TH (1996). The luteinizing hormone chorionic gonadotropin receptor has distinct transmembrane conductors for cAMP and inositol phosphate signals. J. Biol. Chem..

[16] Han SW, Lei ZM, Rao CV (1999). Treatment of human endometrial stromal cells with chorionic gonadotropin promotes their morphological and functional differentiation into decidua. Mol. Cell. Endocrinol..

[17] Licht P, von Wolff M, Berkholz A, Wildt L (2003). Evidence for cycle-dependent expression of full-length human chorionic gonadotropin/luteinizing hormone receptor mRNA in human endometrium and decidua. Fertil. Steril..

[18] Srisuparp S (2003). Signal transduction pathways activated by chorionic gonadotropin in the primate endometrial epithelial cells. Biol. Reprod..

[19] Perrier d’Hauterive S (2004). Human chorionic gonadotropin and growth factors at the embryonic-endometrial interface control leukemia inhibitory factor (LIF) and interleukin 6 (IL-6) secretion by human endometrial epithelium. Hum. Reprod..

[20] Banerjee P, Sapru K, Strakova Z, Fazleabas AT (2009). Chorionic gonadotropin regulates prostaglandin E synthase via a phosphatidylinositol 3-kinase-extracellular regulatory kinase pathway in a human endometrial epithelial cell line: Implications for endometrial responses for embryo implantation. Endocrinology.

[21] Evans J, Salamonsen LA (2013). Too much of a good thing? Experimental evidence suggests prolonged exposure to hCG is detrimental to endometrial receptivity. Hum. Reprod..

[22] Bernardini L, Moretti-Rojas I, Brush M, Rojas F, Balmaceda J (2013). Failure of hCG/LH receptors to stimulate the transmembrane effector adenylyl cyclase in human endometrium. Adv. Biosci. Biotechnol..

[23] Tapia-Pizarro A (2017). hCG activates Epac-Erk1/2 signaling regulating Progesterone Receptor expression and function in human endometrial stromal cells. Mol. Hum. Reprod..

[24] Sacchi S, Sena P, Degli Esposti C, Lui J, La Marca A (2018). Evidence for expression and functionality of FSH and LH/hCG receptors in human endometrium. J. Assist. Reprod. Genet..

[25] Pakarainen T (2007). Extragonadal LH/hCG action: Not yet time to rewrite textbooks. Mol. Cell. Endocrinol..

[26] Madhra M, Gay E, Fraser HM, Duncan WC (2004). Alternative splicing of the human luteal LH receptor during luteolysis and maternal recognition of pregnancy. Mol. Hum. Reprod..

[27] Minegishi T (1997). Expression of luteinizing hormone human chorionic gonadotrophin (LH/HCG) receptor mRNA in the human ovary. Mol. Hum. Reprod..

[28] Stewart EA, Sahakian M, Rhoades A, Van Voorhis BJ, Nowak RA (1999). Messenger ribonucleic acid for the gonadal luteinizing hormone human chorionic gonadotropin receptor is not present in human endometrium. Fertil. Steril..

[29] Chrusciel M, Ponikwicka-Tyszko D, Wolczynski S, Huhtaniemi I, Rahman NA (2019). Extragonadal FSHR expression and function-is it real?. Front. Endocrinol..

[30] Tang BQ, Gurpide E (1993). Direct effect of gonadotropins on decidualization of human endometrial stromal cells. J. Steroid Biochem. Mol. Biol..

[31] Chatterjee A, Jana NR, Bhattacharya S (1997). Stimulation of cyclic AMP, 17 beta-oestradiol and protein synthesis by human chorionic gonadotrophin in human endometrial cells. Hum. Reprod..

[32] Kasahara K (2001). The role of human chorionic gonadotropin on decidualization of endometrial stromal cells in vitro. J. Clin. Endocrinol. Metab..

[33] Kajihara T (2011). Human chorionic gonadotropin confers resistance to oxidative stress-induced apoptosis in decidualizing human endometrial stromal cells. Fertil. Steril..

[34] Macosko EZ (2015). Highly parallel genome-wide expression profiling of individual cells using nanoliter droplets. Cell.

[35] Garcia-Alonso L (2021). Mapping the temporal and spatial dynamics of the human endometrium in vivo and in vitro. Nat. Genet..

[36] Kosaka K (2002). Human chorionic gonadotropin (HCG) activates monocytes to produce interleukin-8 via a different pathway from luteinizing hormone/HCG receptor system. J. Clin. Endocrinol. Metab..

[37] Simpson DZ, Hitchen PG, Elmhirst EL, Taylor ME (1999). Multiple interactions between pituitary hormones and the mannose receptor. Biochem. J..

[38] Kane N, Kelly R, Saunders PTK, Critchley HOD (2009). Proliferation of uterine natural killer cells is induced by human chorionic gonadotropin and mediated via the mannose receptor. Endocrinology.

[39] Mi YL, Lin A, Fiete D, Steirer L, Baenziger JU (2014). Modulation of mannose and asialoglycoprotein receptor expression determines glycoprotein hormone half-life at critical points in the reproductive cycle. J. Biol. Chem..

[40] Ku SY (2002). Effect of gonadotropins on human endometrial stromal cell proliferation in vitro. Arch. Gynecol. Obstet..

[41] Jasinska A, Strakova Z, Szmidt M, Fazleabas AT (2006). Human chorionic gonadotropin and decidualization in vitro inhibits cytochalasin-D-induced apoptosis in cultured endometrial stromal fibroblasts. Endocrinology.

[42] Licht P, Russu V, Wildt L (2001). On the role of human chorionic gonadotropin (hCC) in the embryo-endometrial microenvironment: Implications for differentiation and implantation. Semin. Reprod. Med..

[43] Sherwin JRA (2007). Identification of novel genes regulated by chorionic gonadotropin in baboon endometrium during the window of implantation. Endocrinology.

[44] Mika K (2021). Evolutionary transcriptomics implicates new genes and pathways in human pregnancy and adverse pregnacy outcomes. eLIFE.

[45] Wagner GP, Kin K, Lynch VJ (2012). Measurement of mRNA abundance using RNA-seq data: RPKM measure is inconsistent among samples. Theory Biosci..

[46] Wagner GP, Kin K, Lynch VJ (2013). A model based criterion for gene expression calls using RNA-seq data. Theory Biosci..

[47] Kaur H (2019). Single-cell profiling reveals heterogeneity and functional patterning of GPCR expression in the vascular system. Nat. Commun..

[48] Tischner D (2017). Single-cell profiling reveals GPCR heterogeneity and functional patterning during neuroinflammation. JCI Insight.

[49] Menon KMJ, Munshi UM, Clouser CL, Nair AK (2004). Regulation of luteinizing hormone/human chorionic gonadotropin receptor expression: A perspective. Biol. Reprod..

[50] Tao YX, Johnson NB, Segaloff DL (2004). Constitutive and agonist-dependent self-association of the cell surface human lutropin receptor. J. Biol. Chem..

[51] Lei Y (2007). Constitutively-active human LH receptors are self-associated and located in rafts. Mol. Cell. Endocrinol..

[52] Roess DA, Horvat RD, Munnelly H, Barisas BG (2000). Luteinizing hormone receptors are self-associated in the plasma membrane. Endocrinology.

[53] Horvat RD, Roess DA (2000). Desensitized LH receptors are self-associated in large, slowly diffusing complexes. Biol. Reprod..

[54] Smith SML (2006). Luteinizing hormone receptors translocate to plasma membrane microdomains after binding of human chorionic gonadotropin. Endocrinology.

[55] Zhang ML, Feng XY, Guan RB, Hebert TE, Segaloff DL (2009). A cell surface inactive mutant of the human lutropin receptor (hLHR) attenuates signaling of wild-type or constitutively active receptors via heterodimerization. Cell. Signal..

[56] Feng XY, Zhang ML, Guan RB, Segaloff DL (2013). Heterodimerization between the lutropin and follitropin receptors is associated with an attenuation of hormone-dependent signaling. Endocrinology.

[57] Zhu X, Gilbert S, Birnbaumer M, Birnbaumer L (1994). Dual signaling potential is common among g(s)-coupled receptors and dependent on receptor density. Mol. Pharmacol..

[58] Donadeu FX, Ascoli M (2005). The differential effects of the gonadotropin receptors on aromatase expression in primary cultures of immature rat granulosa cells are highly dependent on the density of receptors expressed and the activation of the inositol phosphate cascade. Endocrinology.

[59] Casarini L, Reiter E, Simoni M (2016). beta-arrestins regulate gonadotropin receptor-mediated cell proliferation and apoptosis by controlling different FSHR or LHCGR intracellular signaling in the hGL5 cell line. Mol. Cell. Endocrinol..

[60] Gilioli L, Marino M, Simon M, Cassarini L (2017). The regulation of LHCGR-dependent signaling is linked to circadian gene expression. Endocr. Abstracts.

[61] Kishi H, Kitahara Y, Imai F, Nakao K, Suwa H (2018). Expression of the gonadotropin receptors during follicular development. Reprod. Med. Biol..

[62] Nair AK, Kash JC, Peegel H, Menon KMJ (2002). Post-transcriptional regulation of luteinizing hormone receptor mRNA in the ovary by a novel mRNA-binding protein. J. Biol. Chem..

[63] Kash JC, Menon KMJ (1998). Identification of a hormonally regulated luteinizing hormone human chorionic gonadotropin receptor mRNA binding protein: Increased mRNA binding during receptor down-regulation. J. Biol. Chem..

[64] Sposini S, Hanyaloglu AC (2018). Driving gonadotrophin hormone receptor signalling: The role of membrane trafficking. Reproduction.

[65] Johnson GP, Jonas KC (2019). Mechanistic insight into how gonadotropin hormone receptor complexes direct signaling†. Biol. Reprod..

[66] Fiddes JC, Goodman HM (1980). The cDNA for the beta-subunit of human chorionic-gonadotropin suggests evolution of a gene by readthrough into the 3'-untranslated region. Nature.

[67] Maston GA, Ruvolo M (2002). Chorionic gonadotropin has a recent origin within primates and an evolutionary history of selection. Mol. Biol. Evol..

[68] Riccetti L (2017). Human luteinizing hormone and chorionic gonadotropin display biased agonism at the LH and LH/CG receptors. Sci. Rep..

[69] Fournier T (2016). Human chorionic gonadotropin: Different glycoforms and biological activity depending on its source of production. Annales D Endocrinologie.

[70] Kovalevskaya G, Birken S, Kakuma T, O'Connor JF (1999). Early pregnancy human chorionic gonadotropin (hCG) isoforms measured by an immunometric assay for choriocarcinoma-like hCG. J. Endocrinol..

[71] Riccetti L (2017). Heterogeneous hCG and hMG commercial preparations result in different intracellular signalling but induce a similar long-term progesterone response in vitro. Mol. Hum. Reprod..

[72] Koistinen H (2019). Hyperglycosylated hCG activates LH/hCG-receptor with lower activity than hCG. Mol. Cell. Endocrinol..

[73] Kajihara T (2011). Differential effects of urinary and recombinant chorionic gonadotropin on oxidative stress responses in decidualizing human endometrial stromal cells. Placenta.

[74] Cole LA (2012). hCG, five independent molecules. Clin. Chim. Acta.

[75] Schafer A, Pauli G, Friedmann W, Dudenhausen JW (1992). Human choriogonadotropin (hCG) and placental-lactogen (HPL) inhibit interleukin-2 (IL-2) and increase interleukin-1-beta (IL-1-beta), interleukin-6 (IL-6) and tumor-necrosis-factor (TNF-alpha) expression in monocyte cell-cultures. J. Perinat. Med..

[76] Yousefi S (1993). The effect of gonadotropins on the production of human interferon-gamma by mononuclear-cells. J. Interferon Res..

[77] Komorowski J, Gradowski G, Stepien H (1997). Effects of hCG and beta-hCG on IL-2 and sIL-2R secretion from human peripheral blood mononuclear cells: A dose-response study in vitro. Immunol. Lett..

[78] McCoy DE, Haig D (2020). Embryo selection and mate choice: Can ‘honest signals’ be trusted?. Trends Ecol. Evol..

[79] Lucas ES (2016). Loss of endometrial plasticity in recurrent pregnancy loss. Stem Cells.

[80] Muter J (2018). The glycosyltransferase EOGT regulates adropin expression in decidualizing human endometrium. Endocrinology.

[81] Diniz-da-Costa M (2021). Characterization of highly proliferative decidual precursor cells during the window of implantation in human endometrium. Stem Cells.

[82] Megill C (2021). cellxgene: A performant, scalable exploration platform for high dimensional sparse matrices. bioRxiv.

[83] Barros F, Brosens J, Brighton P (2016). Isolation and primary culture of various cell types from whole human endometrial biopsies. Bio-Protocol.

[84] Jean-Alphonse F (2014). Spatially restricted G protein-coupled receptor activity via divergent endocytic compartments. J. Biol. Chem..

[85] Han SW, Lei ZM, Rao CV (1996). Up-regulation of cyclooxygenase-2 gene expression by chorionic gonadotropin during the differentiation of human endometrial stromal cells into decidua. Endocrinology.

[86] Han SW, Lei ZM, Rao CV (1997). Homologous down-regulation of luteinizing hormone chorionic gonadotropin receptors by increasing the degradation of receptor transcripts in human uterine endometrial stromal cells. Biol. Reprod..

[87] Salker M (2010). Natural selection of human embryos: Impaired decidualization of endometrium disables embryo-maternal interactions and causes recurrent pregnancy loss. PLoS ONE.

[88] Fluhr H (2006). Human chorionic gonadotropin inhibits insulin-like growth factor-binding protein-1 and prolactin in decidualized human endometrial stromal cells. Fertil. Steril..

[89] Berndt S (2006). Angiogenic activity of human chorionic gonadotropin through LH receptor activation on endothelial and epithelial cells of the endometrium. FASEB J..

[90] Paiva P (2011). Human chorionic gonadotrophin regulates FGF2 and other cytokines produced by human endometrial epithelial cells, providing a mechanism for enhancing endometrial receptivity. Hum. Reprod..

