# Supplementary material for: Expression and function of the luteinizing hormone choriogonadotropin receptor in human endometrial stromal cells
Source: Sci Rep. 2022 May 21;12:8624. doi: 10.1038/s41598-022-12495-9 (PMC9124191; doi:10.1038/s41598-022-12495-9)

Supplementary Information

Expression and function of the luteinizing hormone choriogonadotropin receptor in human endometrial stromal cells

Mann ON, Kong C-S, Lucas ES, Brosens JJ, Hanyaloglu AC and Brighton PJ

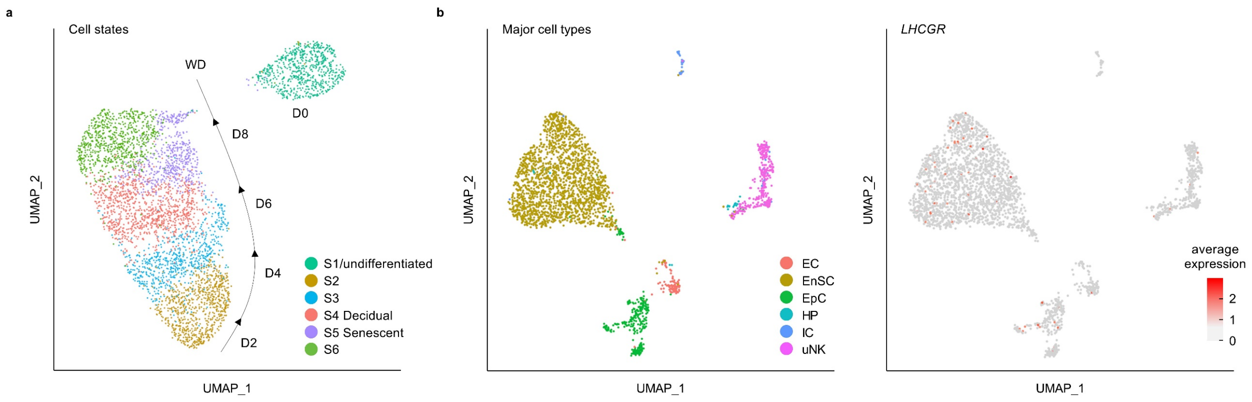

Figure Supplementary S1. Transcriptional clustering of endometrial stromal cells. UMAP (uniform manifold approximation and projection) plots from scRNA-seq analysis of undifferentiated (day 0) EnSC and those treated with C+M for up to 8 days before withdrawal (WD) (GEO GSE127918). Cells are colour coded according to transcriptional states (S1-6). B) UMAP plots from scRNA-seq data of endometrial biopsies (GEO GSE127918), colour coded to identify major endometrial cell types (endothelial (EC), stromal cells (EnSC), epithelial cells (EpC), highly proliferative (HP), immune (IC) and uterine natural killer (uNK) cells) Data adapted from Lucas *et al*, 2020 with permission [3]. C) UMAP plots from the same data set depicting *LHCGR* expression across cell types.

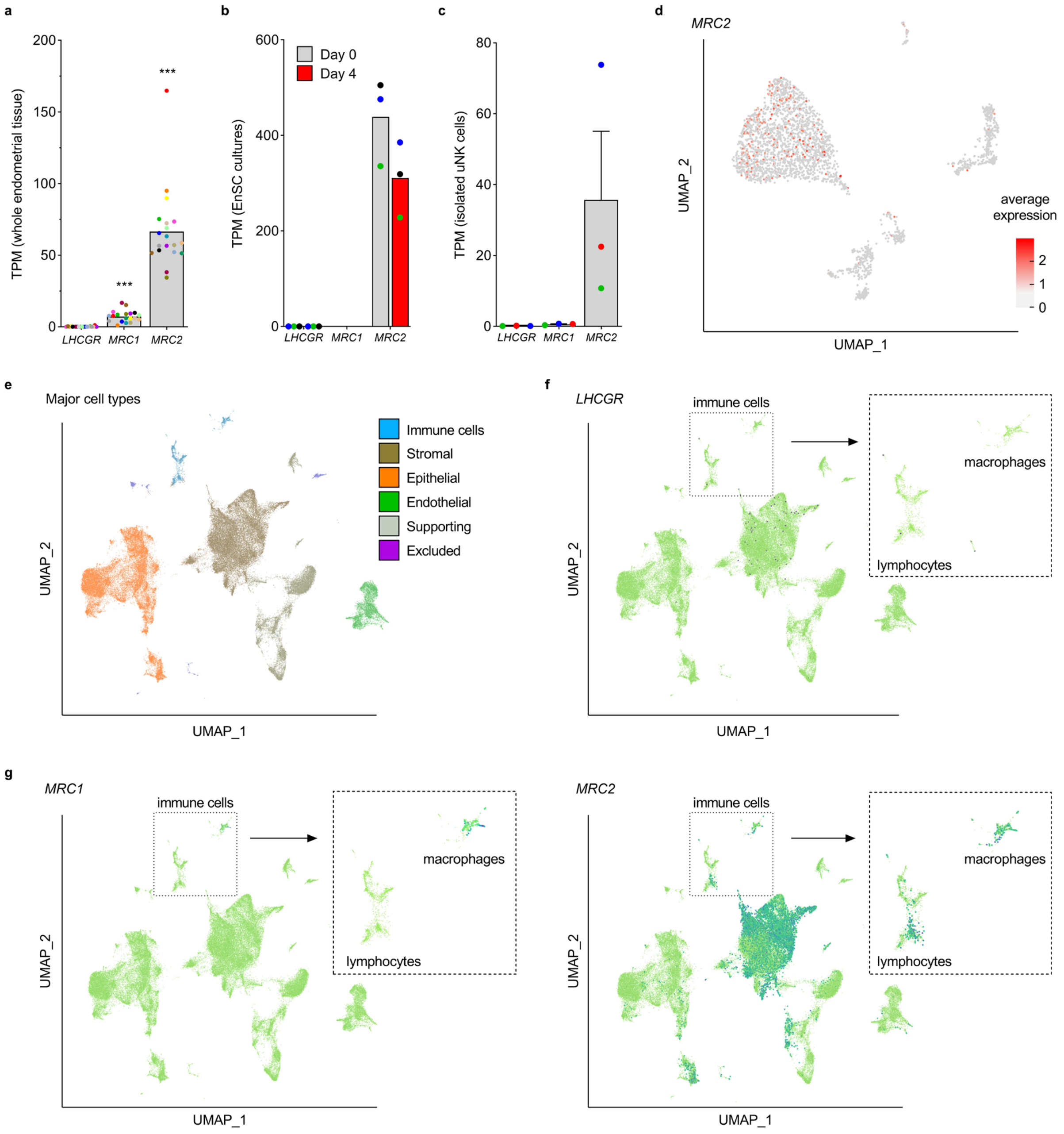

Figure Supplementary S2. Endometrial mannose receptor expression A) Comparison of *LHCGR* expression with the mannose receptor C-type 1 (*MRC1*) and 2 (*MRC2*) in bulk RNA-seq data from whole endometrial tissues (GEO GSE65102). Data points from individual patients are colour matched and shown together with bars denoting mean, n= 20. *** denotes *P*<0.001 by ANOVA and Dunnett's multiple comparison using *LHCGR* as the comparator. B) Expression of *LHCGR*, *MRC1* and *MRC2* in bulk RNA-seq data from undifferentiated EnSC (day 0) and cells decidualized with 8-br-cAMP and MPA (C+M) for 4 days (GEO

GSE104721). C) Expression of *LHCGR*, *MRC1* and *MRC2* in bulk RNA-seq data from isolated uNK cells (GEO GSE1592660). D) UMAP plot depicting *MRC2* expression across different endometrial cells (GEO GSE127918). Refer to Fig. S1B for cell types. *MRC1* was not expressed. E) UMAP plot from scRNA-seq data identifying major cell types within whole endometrial biopsies [36]. F) UMAP plot depicting *LHCGR* expression across endometrial cells. G) UMAP plot depicting *MRC1* (left panel) and *MRC2* (right panel) expression within the same dataset. Inserts highlight the expression of *LHCGR*, *MRC1* and *MRC2* within immune cells.

Table S1. Patient demographics

Endometrial biopsies used for EnSC cultures				
**Figure**	**(n)**	**Age**	**BMI**	**Day of cycle (LH+)**
**2E**	13	36 (33.75-37)	23.5 (19-28.25)	9 (8-10)
**3A**	3	39 (37-39.5)	26 (20-26.25)	8 (7-8)
**3B**	3	38 (33-39.5)	21 (20-23.5)	7 (5-8)
**3C**	3	36 (30-39)	23 (22-23)	11 (8-11)
**4A-B**	3	35 (35-38)	23 (22-23.75)	9 (8-9.5)
**4C-D**	3	39 (37-39.5)	26 (20-26.25)	8 (7-8)
**5B and D**	3	35 (34-35.5)	25 (25-26)	8 (7-8.5)
**5C**	9	38 (34-41)	25 (22-27)	8 (7-9)

All data are median (interquartile range, Q1-Q3).
LH+: Days since the pre-ovulatory Luteinising Hormone surge

Original uncropped western blots for figure 2d. HEK-WT and HEK-LHCGR cells were stimulated with 10nM hCG for time points indicated and levels of phospho-ERK and Total ERK were determined by western blotting. The set of blots on the left represent a lower exposure to define and separate individual bands, as shown in figure 2d. The set on the right was captured in parallel at high exposure to show the edge of blots in compliance with digital image and integrity policies.

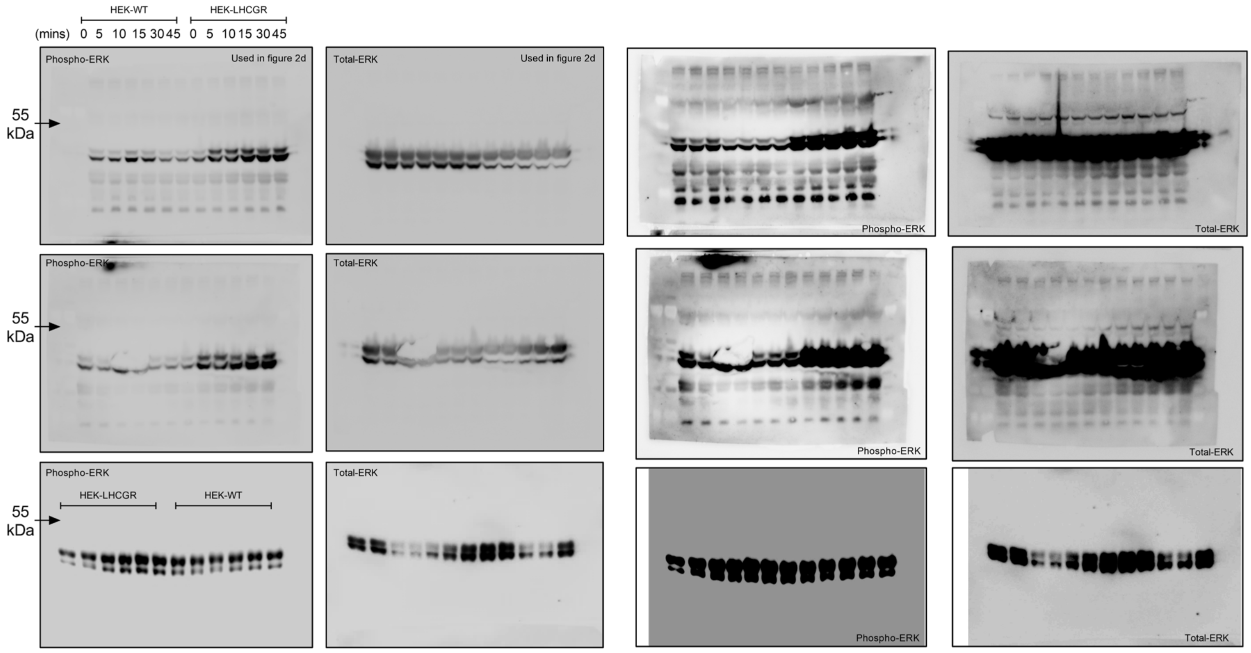

Original uncropped western blots for figure 2d. HEK-WT and HEK-LHCGR cells were stimulated with 10nM LH for time points indicated and levels of phospho-ERK and Total ERK were determined by western blotting. The set of blots on the left represent a lower exposure to define and separate individual bands, as shown in figure 2d. The set on the right was captured in parallel at high exposure to show the edge of blots in compliance with digital image and integrity policies.

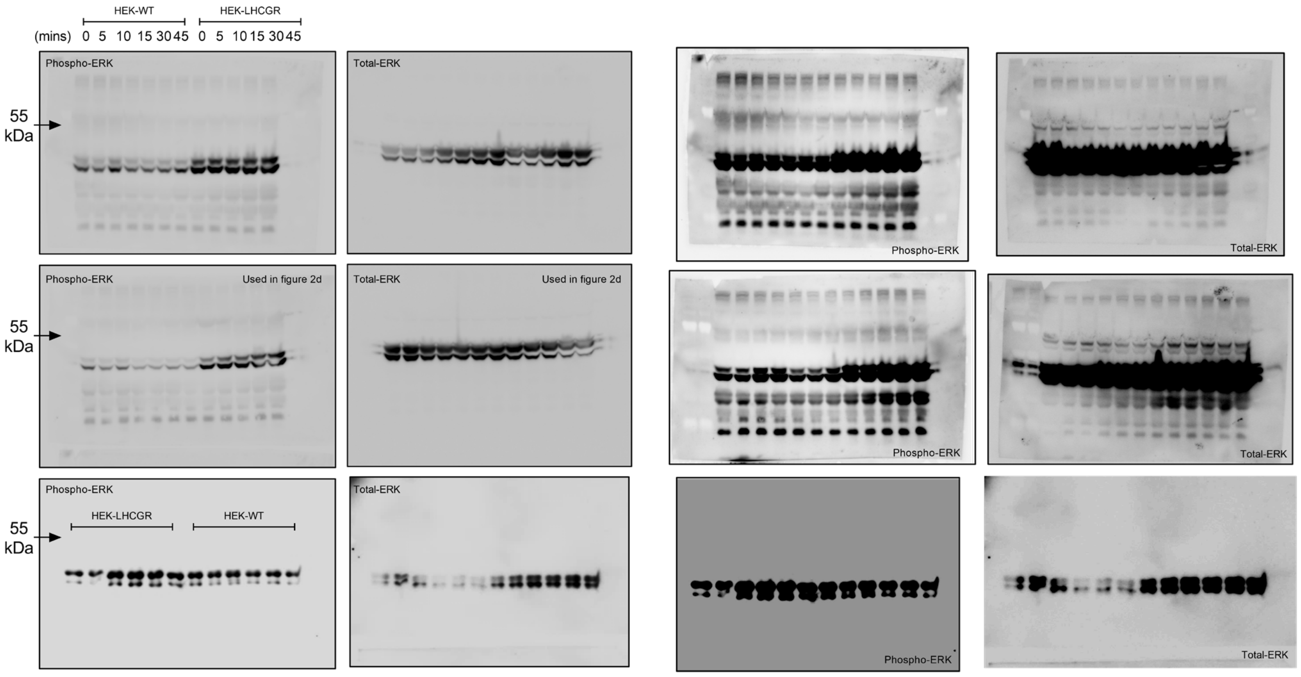

Original uncropped western blots for figure 3c. Undifferentiated and decidualized EnSC were stimulated with 10nM hCG for time points indicated and levels of phospho-ERK and Total ERK were determined by western blotting. The set of blots on the left represent a lower exposure to define and separate individual bands, as shown in figure 3c. The set on the right was captured in parallel at high exposure to show the edge of blots in compliance with digital image and integrity policies.

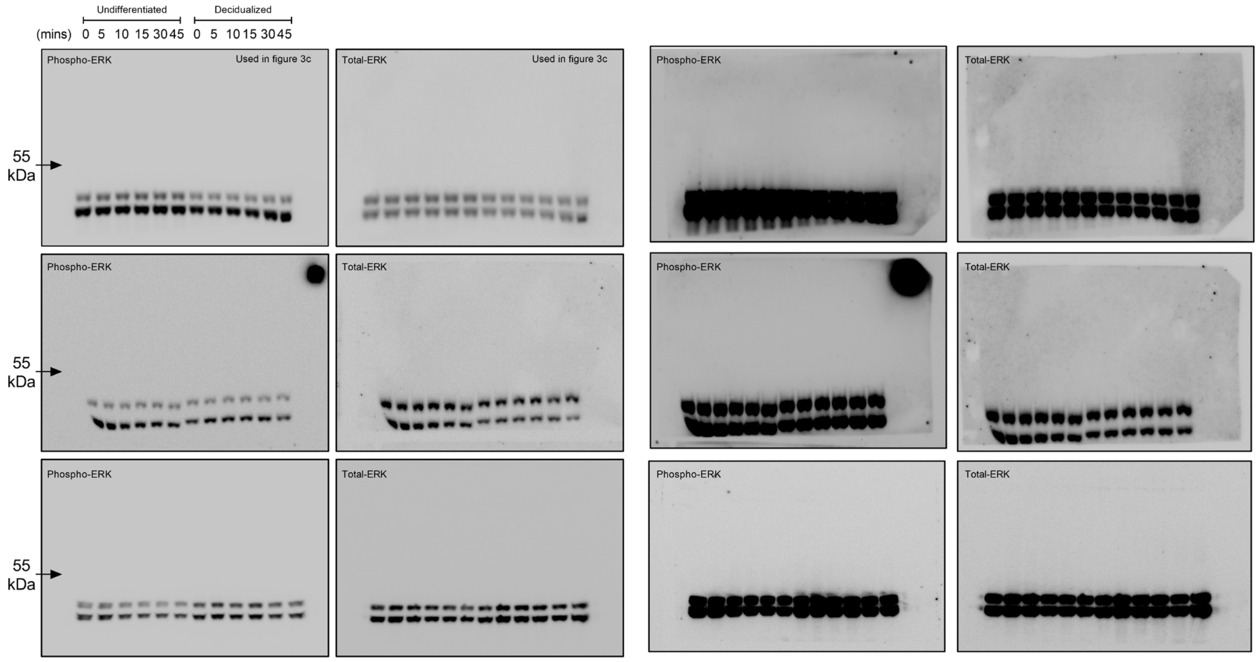

Original uncropped western blots for figure 3c. Undifferentiated and decidualized EnSC were stimulated with 10nM LH for time points indicated and levels of phospho-ERK and Total ERK were determined by western blotting. The set of blots on the left represent a lower exposure to define and separate individual bands, as shown in figure 3c. The set on the right was captured in parallel at high exposure to show the edge of blots in compliance with digital image and integrity policies.

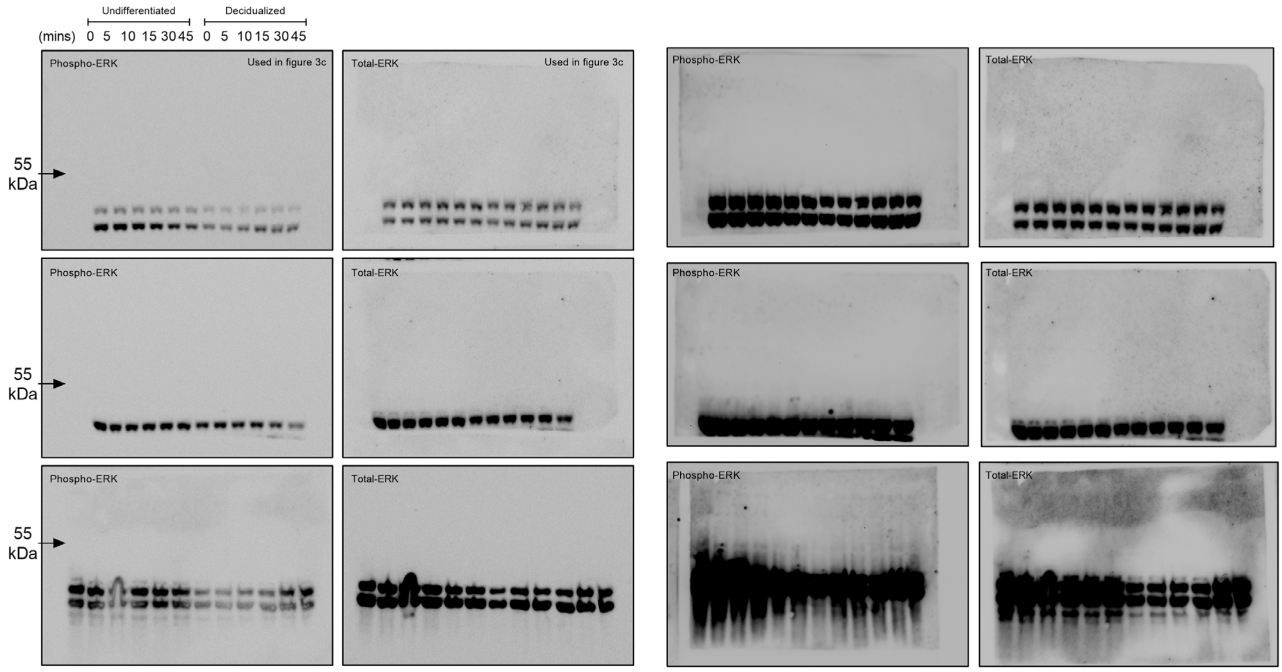

Supplement: Supplementary file 1 — Supplementary Information. [file 41598_2022_12495_MOESM1_ESM.pdf]
